# Network analysis of physical exercise, sleep quality, and psychological distress among Chinese university students

**DOI:** 10.1371/journal.pone.0362609

**Published:** 2026-11-12

**Authors:** Jingbo Wang, Yuanyuan Ma, Tong Han, Juan Wang

**Affiliations:** 1 Henan University, Kaifeng, Henan, China; 2 School of Sports Science, Jishou University, Jishou, Hunan, China; 3 Gdansk University of Physical Education and Sport, Gdańsk, Pomeranian Voivodeship, The Republic of Poland; 4 School of General Education, Shandong Huayu University of Technology, Dezhou, Shandong, China; Kyoto University: Kyoto Daigaku, JAPAN

## Abstract

**Objective:**

Physical exercise, sleep quality, and psychological distress may be interconnected among university students, but their dimension-specific associations remain unclear. This study examined their network structure and compared male and female networks.

**Methods:**

A convenience sample of 1,600 university students from two higher-education institutions in central China was surveyed from 16 March to 29 May 2026. Physical exercise, sleep quality, and psychological distress were assessed using the Physical Activity Rating Scale, Pittsburgh Sleep Quality Index, and 21-item Depression Anxiety Stress Scales. A 12-node regularized partial correlation network was estimated. Expected influence, bridge expected influence, bootstrap procedures, and network comparison tests were used.

**Results:**

The network retained 27 non-zero edges, with stronger connections mainly within domains. Daytime dysfunction–stress had the largest positive cross-domain edge-weight point estimate, whereas exercise frequency–stress and exercise duration–subjective sleep quality were prominent negative connections. Stress showed relatively high expected influence and positive bridge expected influence point estimates, although bootstrap difference tests did not clearly distinguish these estimates from those of several other nodes. No gender-related differences were detected in global strength, network structure, or individual edges after multiple-comparison correction, while supplementary analyses indicated limited detection capability.

**Conclusions:**

Cross-domain associations among physical exercise, sleep quality, and psychological distress were concentrated in a limited number of dimensions, particularly stress, daytime dysfunction, exercise frequency, and exercise duration. Stress and daytime dysfunction showed relatively prominent sleep–psychological distress associations, whereas higher exercise frequency and longer exercise duration were associated with lower stress and fewer subjective sleep problems, respectively. No gender-related network differences were detected, but this does not establish network equivalence. These cross-sectional findings identify dimension-specific associations but cannot establish causal relationships or intervention targets.

## Introduction

The university period is a critical stage during which psychological problems commonly emerge and may influence subsequent development. The World Health Organization World Mental Health International College Student Survey showed that 31.4% of first-year university students screened positive for at least one common mental disorder within the past 12 months [1]. Among students who screened positive for at least one mental disorder in the past 12 months, 42.9% reported severe role impairment in at least one domain, including household management, university-related academic activities, intimate relationships, or social life [2]. Therefore, mental health problems among university students are not only manifested as symptoms of depression, anxiety, and stress but may also affect their academic adjustment and daily functioning. University students are also situated in the developmental period of emerging adulthood, during which increasing autonomy, identity exploration, and changing social and academic demands may coincide with greater psychological vulnerability [3]. Recent network evidence further suggests that psychological distress in university students is embedded within broader systems of psychological vulnerabilities and resources rather than occurring in isolation [4]. In China, this developmental transition takes place within a distinctive campus context that includes shared residential environments and substantial academic demands, both of which may shape students’ exercise behaviors, sleep patterns, and psychological well-being [5,6].

Sleep and physical exercise are modifiable lifestyle factors that are closely associated with mental health among university students. An updated meta-analysis including 232 studies and 495,641 Chinese university students showed that the pooled prevalence of sleep problems was 26.4%; however, substantial heterogeneity existed across studies, suggesting considerable individual and contextual variations in sleep problems among university students [7]. Sleep quality involves multiple components, including subjective sleep quality, sleep latency, sleep duration, sleep efficiency, sleep disturbances, and daytime dysfunction [8]. Physical exercise similarly encompasses different characteristics, including intensity, duration per session, and participation frequency [9]. Meta-analyses of prospective studies have shown that higher levels of physical activity are associated with lower risks of subsequent depression and anxiety onset [10,11]. Physical exercise may also improve some sleep indicators; however, its effects are influenced by exercise type, intensity, duration, and measurement methods [12,13]. Both physical exercise and sleep quality have multidimensional characteristics. Different exercise components and sleep manifestations may exhibit distinct association patterns with psychological distress. Therefore, further investigation of the relationships among these three constructs at the specific dimensional level is warranted.

Physical exercise, sleep, and psychological distress may constitute an interconnected health system. For example, insufficient exercise may co-occur with more severe sleep problems and psychological distress, while symptoms of depression, anxiety, and stress may also be associated with students’ difficulties in maintaining regular exercise and stable sleep schedules. Traditional analyses typically use total scale scores and examine relationships among a limited number of variables based on predefined directions, making it difficult to simultaneously capture conditional associations among multiple exercise dimensions, sleep components, and psychological distress dimensions. Network analysis can represent these dimensions as nodes and represent conditional associations after controlling for other nodes in the network as edges, thereby identifying nodes with relatively stronger associations and bridge nodes that connect different theoretical domains [14]. Centrality and bridge centrality are descriptive summaries of the estimated network structure and should be interpreted primarily as hypothesis-generating indices rather than as direct evidence of causal importance or intervention effects [15–17].

In recent years, network analysis has been increasingly applied in research on sleep and mental health among university students. Tao et al. constructed a network of depression, anxiety, and sleep symptoms and found that sleep dissatisfaction, poor sleep quality, and uncontrollable worry exhibited higher centrality [18]. He et al. further found that subjective sleep quality, daytime dysfunction, and fatigue occupied important positions in the anxiety–sleep network [19]. A limited number of studies have begun to incorporate physical exercise into the network framework. Yang et al. constructed a network of physical activity and sleep disturbances and further examined their associations with depression and anxiety using separate flow networks. They found that daytime dysfunction, exercise intensity, and exercise frequency played relatively prominent roles in the network [20]. Feng et al. further investigated the network structure and gender differences between sleep problems and physical exercise among Chinese university students [21]. However, these studies did not simultaneously represent physical exercise dimensions, sleep components, and depression, anxiety, and stress as nodes within a single network. More importantly, stress has received comparatively limited attention in existing physical exercise–sleep network research. Stress warrants separate consideration because it reflects experiences such as tension, irritability, and difficulty relaxing and represents a distinguishable dimension of psychological distress [22]. Exercise participation may covary with stress through patterns of psychological recovery and tension regulation [23], whereas stress-related cognitive arousal may be associated with poorer sleep; conversely, poor sleep may also contribute to subsequent stress [24]. These reciprocal cross-domain associations provide a rationale for examining stress together with exercise and sleep rather than treating psychological distress solely in terms of depression and anxiety.

Based on this, the present study constructed a physical exercise–sleep quality–psychological distress network in a sample of Chinese university students. Physical exercise intensity, duration, and frequency; subjective sleep quality, sleep latency, sleep duration, sleep efficiency, sleep disturbances, and daytime dysfunction; as well as depression, anxiety, and stress were simultaneously included in the analysis. This study aimed to: (1) examine the within-domain and between-domain conditional associations among the three domains; (2) identify nodes with relatively stronger associations and bridge nodes connecting different domains within the network; and (3) compare the overall network structure, global strength, and specific edge weights between males and females. The study also evaluated the accuracy, stability, and robustness of the findings through bootstrap procedures and multiple sensitivity analyses, aiming to provide evidence for understanding the dimension-specific associations among physical exercise, sleep problems, and psychological distress among university students. Based on previous evidence, we expected physical exercise dimensions to show predominantly negative cross-domain associations with sleep problems and psychological distress, whereas poorer sleep components were expected to show predominantly positive associations with psychological distress. However, because evidence concerning the specific node-to-node pattern remains limited, the relative prominence of individual edges and bridge nodes was treated as exploratory.

## Methods

### Study design and participants

This study employed a cross-sectional survey design. From 16 March to 29 May 2026, first- and second-year university students were recruited from one general university and one higher vocational college in central China using a convenience sampling method. Data collection at both participating institutions was conducted with institutional permission. The survey was administered uniformly in classrooms using paper-based questionnaires. Faculty members were present only for classroom coordination and did not participate in questionnaire completion, inspect individual responses, or influence students’ decisions regarding participation. Participants completed the questionnaires independently and submitted them on site.

A total of 1,680 questionnaires were distributed. After excluding questionnaires with more than 20% missing data or clear response-pattern abnormalities identified during manual screening, such as mechanically repeated response sequences across multiple consecutive items, 1,600 participants were included in the final analysis, resulting in a usable questionnaire rate of 95.2%. Among them, 674 participants were male and 926 were female; 784 participants (49.0%) were recruited from the general university and 816 (51.0%) from the higher vocational college; 751 (46.9%) were first-year students and 849 (53.1%) were second-year students. The mean age was 18.46 years (SD = 0.89; range = 17–21 years). Eighty participants were younger than 18 years. All participants had complete data for the 12 nodes included in the primary network. This study was approved by the Biomedical Ethics Committee of Jishou University (JSDX-2025–0074). Participation was voluntary, and the confidentiality of participants’ personal information was protected throughout data collection and analysis. Written informed consent was obtained from all adult participants. For participants younger than 18 years, written informed consent was obtained from a parent or legal guardian, together with the participant’s assent.

### Measurement instruments

#### Physical exercise.

*The Physical Activity Rating Scale-3* (PARS-3)*,* developed by Liang Deqing, was used to assess participants’ physical exercise over the past month [25]. The scale consists of three items assessing exercise intensity, duration per exercise session, and exercise frequency, each using five ordered response categories. The conventional PARS-3 exercise amount score is calculated as exercise intensity × (exercise duration − 1) × exercise frequency, yielding a total score ranging from 0 to 100. According to the conventional classification, scores ≤19, 20–42, and ≥43 indicate low, moderate, and high exercise amounts, respectively [26]. In the present study, however, the total exercise amount score and its categorical classification were not used as network nodes because the objective was to examine dimension-specific associations. Instead, exercise intensity, duration, and frequency were retained separately as three physical exercise nodes. The test–retest reliability of the Chinese version of the scale was 0.82.

### Sleep quality

*The Pittsburgh Sleep Quality Index* (PSQI) was used to assess participants’ sleep status over the past month [27]. This study used the Chinese version of the scale translated by Liu Xianchen et al. [28]. The PSQI consists of seven components: subjective sleep quality, sleep latency, sleep duration, sleep efficiency, sleep disturbances, use of sleep medication, and daytime dysfunction. Each component is scored from 0 to 3, with higher scores indicating more severe sleep problems. The seven component scores were summed to obtain a global PSQI score ranging from 0 to 21, with higher scores indicating poorer overall sleep quality; a global score >5 was used to indicate poor sleep quality. Because the use of sleep medication component exhibited a pronounced floor effect, with 97.0% of participants scoring 0, the primary network included the remaining six components, and the use of sleep medication node was reintroduced in the sensitivity analysis. The Cronbach’s α coefficient for the overall PSQI was 0.84. Because the PSQI components were analyzed separately as network nodes, this overall coefficient should not be interpreted as the reliability of each individual component.

### Psychological distress

*The Depression Anxiety Stress Scale-21* (DASS-21) was originally developed by Lovibond et al. in 1995 [29]. This study used the simplified Chinese version of the DASS-21 revised by Gong et al. [30]. The full scale consists of 21 items, with each of the three subscales for depression, anxiety, and stress containing 7 items. All items are rated on a four-point scale ranging from “0” (did not apply) to “3” (applied most of the time). The scores of each subscale are multiplied by 2 to obtain the final subscale scores, with higher scores indicating greater levels of the corresponding emotional state. For descriptive purposes, conventional DASS-21 severity bands were applied to the multiplied subscale scores: depression, 0–9 normal, 10–13 mild, 14–20 moderate, 21–27 severe, and ≥28 extremely severe; anxiety, 0–7, 8–9, 10–14, 15–19, and ≥20, respectively; and stress, 0–14, 15–18, 19–25, 26–33, and ≥34, respectively. The depression subscale consists of items 3, 5, 10, 13, 16, 17, and 21; the anxiety subscale consists of items 2, 4, 7, 9, 15, 19, and 20; and the stress subscale consists of items 1, 6, 8, 11, 12, 14, and 18. Depression, anxiety, and stress were included as separate network nodes. The Cronbach’s α coefficient of the scale was 0.89, and the Cronbach’s α coefficients of the depression, anxiety, and stress subscales were 0.77, 0.79, and 0.76, respectively. McDonald’s ω coefficients for the three subscales were 0.80, 0.78, and 0.81, respectively. A three-factor confirmatory factor analysis using WLSMV estimation showed adequate model fit (χ²(186) = 152.03, P = 0.106, CFI = 1.000, TLI = 1.002, RMSEA = 0.000, SRMR = 0.028).

### Statistical analysis

All analyses were conducted in R version 4.6.1, primarily using qgraph (version 1.9.8), bootnet (version 1.8), networktools (version 1.6.0), NetworkComparisonTest (version 2.2.3), and EGAnet (version 2.4.1). Reporting of the network analyses followed the reporting standards for psychological network analyses in cross-sectional data proposed by Burger et al. [31]. First, the mean, standard deviation, skewness, and kurtosis of each variable were calculated, and Spearman correlation coefficients were used to describe zero-order correlations.

A Gaussian graphical model was used to estimate the network structure. Nodes represented scores of individual dimensions, components, or subscales rather than individual symptoms, and edges represented regularized partial correlations between two nodes after controlling for all other nodes in the network. Accordingly, the network was interpreted as a dimension-level conditional association network rather than as a symptom-interaction model. Positive and negative edges represented positive and negative conditional associations, respectively. Larger absolute edge weights indicated stronger associations.

Considering that the variables included both ordinal categorical and continuous scores, the cor_auto function in the qgraph package was used to estimate the correlation matrix. Based on this matrix, the extended Bayesian information criterion graphical least absolute shrinkage and selection operator (EBICglasso) was applied to estimate the network, with the hyperparameter γ set to 0.50. The full-sample network and male and female networks used the same node order, estimation method, and model parameters, with consistent node layouts and edge weight scales.

One-step Expected Influence (EI) was used to evaluate node centrality. EI represents the signed sum of the edge weights between a node and all adjacent nodes. One-step Bridge Expected Influence (BEI) was used to evaluate the extent to which nodes were connected with other communities. BEI also preserves the direction of edge weights; therefore, the highest positive BEI and the negative BEI with larger magnitudes were examined separately. Absolute bridge strength, defined as the sum of the absolute weights of a node’s cross-community edges, was additionally calculated to characterize the overall magnitude of cross-community connectivity irrespective of direction. Bootstrap difference tests were used to compare EI and BEI between nodes.

A total of 1,000 nonparametric bootstrap iterations were used to estimate the 95% confidence intervals of edge weights and evaluate the precision of edge weight estimates. Non-zero edges in the regularized network were described as retained edges and were not directly equivalent to statistical significance in traditional statistical analyses. A total of 1,000 case-dropping bootstrap iterations were conducted to examine the stability of EI and BEI, with case-dropping proportions ranging from 10% to 75%. Stability was evaluated using the correlation stability coefficient (CS; cor = 0.70), with CS ≥ 0.50 considered acceptable.

The Network Comparison Test (NCT) was used to compare male and female networks, with 1,000 permutations performed. The NCT used the same network-estimation procedure as the primary analysis, with cor_auto followed by EBICglasso (γ = 0.50); the networks were treated as weighted and unpaired, and global strength was based on the sum of absolute edge weights. The tests included invariance of global strength, invariance of network structure, and differences in specific edge weights. Two-sided P < 0.05 was considered statistically significant for tests of global strength and network structure. For edge-level tests, multiple comparison corrections were performed using both the Bonferroni method and the Benjamini–Hochberg false discovery rate method. Differences were considered statistically significant when Bonferroni-adjusted P < 0.05 or BH-FDR-adjusted q < 0.05. To evaluate the robustness of the gender comparison, network accuracy and centrality stability were additionally assessed separately in males and females. Sensitivity analyses further repeated the NCT after matching the female sample size to the male sample size and across 100 repeated matched subsamples. An observed-effect simulation with pooled-null calibration was conducted as a supplementary evaluation of the detection capability of the gender comparison; these simulation results were treated as exploratory rather than as an a priori power analysis (S10 Table).

To examine the robustness of the main findings, the following sensitivity analyses were further conducted: first, the EBIC hyperparameter was adjusted from γ = 0.50 to γ = 1.00; second, the use of sleep medication was included in the network to form a 13-node model; and third, the network was re-estimated using the Spearman correlation matrix of all variables. For the γ = 1.00 model and the Spearman correlation matrix model, edge weights, non-zero edge sets, and EI and BEI rankings were compared. For the 13-node model, EI and BEI rankings of common nodes and major network characteristics were compared.

Finally, to examine whether pooling participants from the two institutions influenced the network structure, each of the 12 network nodes was residualized for institution, grade, and age using linear regression, with institution and grade treated as categorical variables. A covariate-adjusted network was then re-estimated from the Pearson correlation matrix of the residuals using EBICglasso with γ = 0.50, and its edge weights, non-zero edge set, EI, and BEI were compared with those of the primary network.

Node redundancy was assessed using Unique Variable Analysis based on weighted topological overlap (wTO), with 0.25 as the prespecified redundancy threshold. Empirical community structure was examined using exploratory graph analysis with GLASSO estimation and the Walktrap algorithm, with stability evaluated using 500 bootstrap resamples. BEI was additionally recalculated using the empirical communities while retaining the primary network edge-weight matrix; sleep latency, which was unassigned by EGA, was treated as a singleton community.

Edge retention frequency across bootstrap samples was also calculated to assess the stability with which each regularized edge was retained. Bootstrap confidence intervals and edge-difference intervals were interpreted as measures of sampling variability rather than conventional significance tests. Because the edge estimates were regularized, the bootstrap distributions and intervals were also affected by shrinkage toward zero.

## Results

### Descriptive statistics and zero-order correlations

A total of 1,600 university students were included in the final analysis, including 674 males and 926 females. All participants had complete data for the 12 nodes included in the primary network. Descriptive statistics and Spearman zero-order correlations for all variables are presented in Table 1. The mean global PSQI score was 4.11 (SD = 2.13), and 383 participants (23.9%) had a global score >5, indicating poor sleep quality. The distributions of depression, anxiety, and stress across the conventional DASS-21 severity bands are presented in S11 Table. Exercise intensity, duration, and frequency were positively correlated with each other (r = 0.175–0.282, all P < 0.001). All three physical exercise indicators were negatively correlated with subjective sleep quality, sleep latency, depression, anxiety, and stress (|r| = 0.050–0.204, all P < 0.05). Exercise duration and exercise frequency were both negatively correlated with sleep disturbances and daytime dysfunction (|r| = 0.059–0.118, all P < 0.05). None of the three physical exercise indicators showed statistically significant correlations with sleep duration or sleep efficiency (all P > 0.05). Depression, anxiety, and stress were positively correlated with each other (r = 0.258–0.304, all P < 0.001). Subjective sleep quality, sleep latency, sleep disturbances, and daytime dysfunction were all positively correlated with depression, anxiety, and stress (all P < 0.05).

**Table 1:** Descriptive statistics and zero-order correlations.

Variable	Mean	SD	Skewness	Kurtosis	1	2	3	4	5	6	7	8	9	10	11
1 PE Intensity	2.001	1.218	1.136	0.423											
2 PE Duration	2.911	1.414	0.162	−1.265	0.257^***^										
3 PE Frequency	2.424	1.103	0.979	0.364	0.175^***^	0.282^***^									
4 Subjective Sleep Quality	0.794	0.637	0.467	0.504	−0.056^*^	−0.144^***^	−0.121^***^								
5 Sleep Latency	0.694	0.803	1.006	0.405	−0.054^*^	−0.063^*^	−0.061^*^	0.190^***^							
6 Sleep Duration	0.393	0.528	0.921	0.173	0.005	−0.014	0.007	0.021	0.084^***^						
7 Sleep Efficiency	0.564	0.995	1.627	1.218	−0.014	0.023	−0.030	0.006	0.036	0.144^***^					
8 Sleep Disturbance	0.681	0.594	0.386	0.150	−0.022	−0.061^*^	−0.118^***^	0.198^***^	0.100^***^	0.049	0.025				
9 Day Dysfunction	0.939	0.809	0.657	0.053	−0.029	−0.110^***^	−0.059^*^	0.179^***^	0.042	0.0002	−0.050^*^	0.144^***^			
10 Depression	5.955	7.248	1.545	2.961	−0.106^***^	−0.070^**^	−0.098^***^	0.067^**^	0.067^**^	0.058^*^	0.004	0.166^***^	0.078^**^		
11 Anxiety	6.884	7.282	1.323	2.197	−0.050^*^	−0.081^**^	−0.095^***^	0.081^**^	0.120^***^	0.007	0.009	0.080^**^	0.108^***^	0.258^***^	
12 Stress	7.401	7.983	1.122	1.277	−0.081^**^	−0.120^***^	−0.204^***^	0.180^***^	0.056^*^	0.054^*^	0.026	0.129^***^	0.230^***^	0.304^***^	0.291^***^

Note. N = 1,600. Correlations are Spearman’s rank correlation coefficients. *** p < 0.001; ** p < 0.01; * p < 0.05.

### Overall network structure

The primary network retained 27 non-zero edges out of 66 possible edges, with a network density of 40.9%. The five edges with the largest weights were, in descending order, exercise duration–exercise frequency (edge weight = 0.217), exercise intensity–exercise duration (edge weight = 0.208), depression–stress (edge weight = 0.207), anxiety–stress (edge weight = 0.172), and subjective sleep quality–sleep latency (edge weight = 0.166). Stronger connections were primarily concentrated within their respective domains (see Fig 1 and Table 2).

**Table 2:** Strongest network edges.

Rank	Node 1	Node 2	Community type	Edge weight	Absolute edge weight	Bootstrap 95% CI low	Bootstrap 95% CI high
1	PE Duration	PE Frequency	within PE	0.2172	0.2172	0.1729	0.2757
2	PE Intensity	PE Duration	within PE	0.2080	0.2080	0.1624	0.2740
3	Depression	Stress	within DASS	0.2074	0.2074	0.1630	0.2586
4	Anxiety	Stress	within DASS	0.1716	0.1716	0.1311	0.2209
5	Subjective Sleep Quality	Sleep Latency	within PSQI	0.1655	0.1655	0.1159	0.2243
6	Subjective Sleep Quality	Sleep Disturbance	within PSQI	0.1648	0.1648	0.1130	0.2363
7	Sleep Duration	Sleep Efficiency	within PSQI	0.1582	0.1582	0.0952	0.2476
8	Day Dysfunction	Stress	DASS–PSQI	0.1483	0.1483	0.1076	0.2010
9	Depression	Anxiety	within DASS	0.1464	0.1464	0.1023	0.2089
10	Subjective Sleep Quality	Day Dysfunction	within PSQI	0.1178	0.1178	0.0697	0.1787

**Fig 1:**
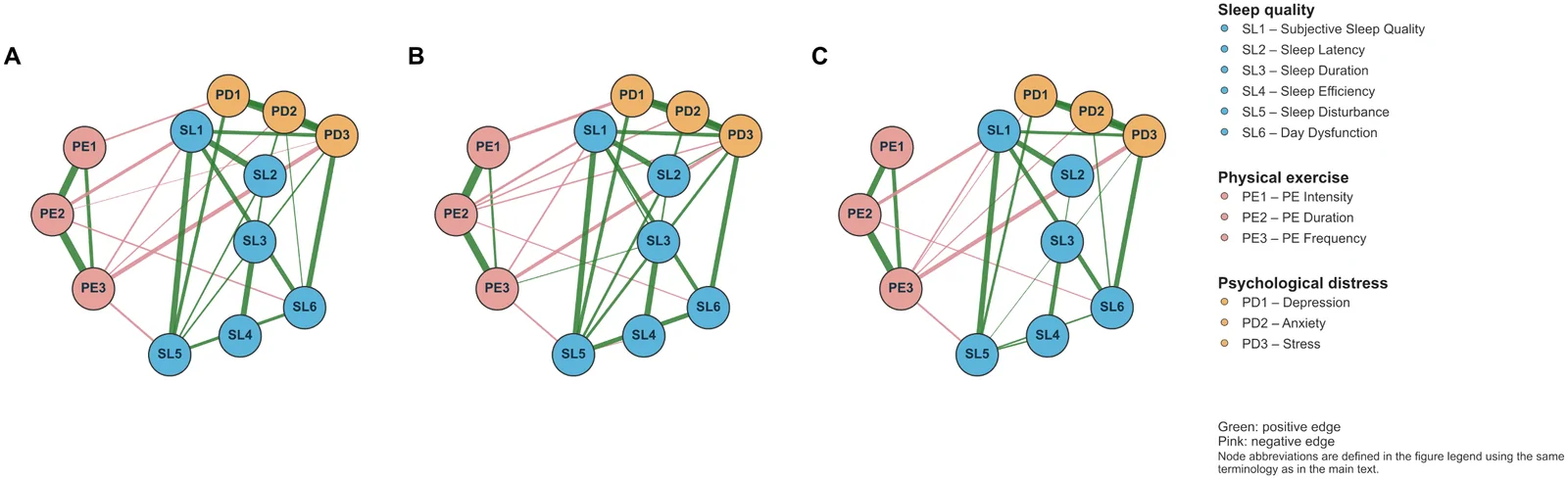
Full-sample, male, and female networks. Note. Full-sample, male, and female networks. Panels A, B, and C represent the full-sample, male, and female networks, respectively. Green edges indicate positive associations, whereas pink edges indicate negative associations; thicker edges represent stronger associations. The three networks use the same node layout and edge-weight scale. Node abbreviations are defined in the figure legend using the same terminology as in the main text.

The primary network retained a total of 14 cross-domain edges. The mean absolute weight of all possible cross-domain edges was 0.017, whereas the mean absolute weight of non-zero cross-domain edges was 0.056. Daytime dysfunction–stress had the largest positive cross-domain edge-weight point estimate (edge weight = 0.148, 95% CI 0.108 to 0.201), followed by subjective sleep quality–stress (edge weight = 0.094, 95% CI 0.044–0.147) and sleep disturbances–depression (edge weight = 0.087, 95% CI 0.040 to 0.147). The prominent negative cross-domain edges were exercise frequency–stress (edge weight = −0.111, 95% CI −0.161 to -0.066) and exercise duration–subjective sleep quality (edge weight = −0.081, 95% CI −0.139 to -0.028). All 66 edges and 27 non-zero edges are presented in S1 and S2 Tables, respectively. Bootstrap difference tests showed that the daytime dysfunction–stress edge was not clearly distinguishable from subjective sleep quality–stress or sleep disturbance–depression (S9 Table).

### Node centrality and bridge centrality

In the one-step Expected Influence (EI) analysis, the stress node had the highest point estimate of EI (EI = 0.541), followed by subjective sleep quality (EI = 0.424) and depression (EI = 0.401) (see Fig 2 and Table 3). Bootstrap difference tests showed that the EI difference between stress and subjective sleep quality was 0.117, with a 95% bootstrap interval of −0.053 to 0.289; the difference between stress and depression was 0.140, with a 95% bootstrap interval of −0.001 to 0.293. Both intervals included zero. Therefore, although stress showed the highest EI point estimate, its centrality was not clearly distinguishable from that of subjective sleep quality and depression. The EI of stress was higher than those of anxiety and daytime dysfunction, and the corresponding bootstrap difference intervals did not include zero.

**Table 3:** Expected influence, bridge expected influence, and absolute bridge strength.

Node	Community	EI	EI rank	BEI	BEI rank	Absolute bridge strength
PE Intensity	physical exercise	0.2570	9	−0.0399	10	0.0399
PE Duration	physical exercise	0.3127	7	−0.1125	11	0.1125
PE Frequency	physical exercise	0.0908	12	−0.2154	12	0.2154
Subjective Sleep Quality	sleep quality	0.4238	2	−0.0244	9	0.2115
Sleep Latency	sleep quality	0.2788	8	0.0387	5	0.0387
Sleep Duration	sleep quality	0.1988	10	0.0000	7	0.0000
Sleep Efficiency	sleep quality	0.1582	11	0.0000	7	0.0000
Sleep Disturbance	sleep quality	0.3488	5	0.0728	3	0.1665
Day Dysfunction	sleep quality	0.3261	6	0.1311	2	0.1916
Depression	psychological distress	0.4010	3	0.0471	4	0.1269
Anxiety	psychological distress	0.3491	4	0.0311	6	0.0723
Stress	psychological distress	0.5412	1	0.1622	1	0.3868

Note. EI = expected influence; BEI = bridge expected influence. Both EI and BEI retain the signs of edge weights. Absolute bridge strength is the sum of the absolute weights of all cross-community edges connected to a node and therefore reflects the overall magnitude of cross-community connectivity irrespective of direction. Negative BEI values indicate predominantly negative cross-community connections and should not be interpreted as causal protection.

**Fig 2:**
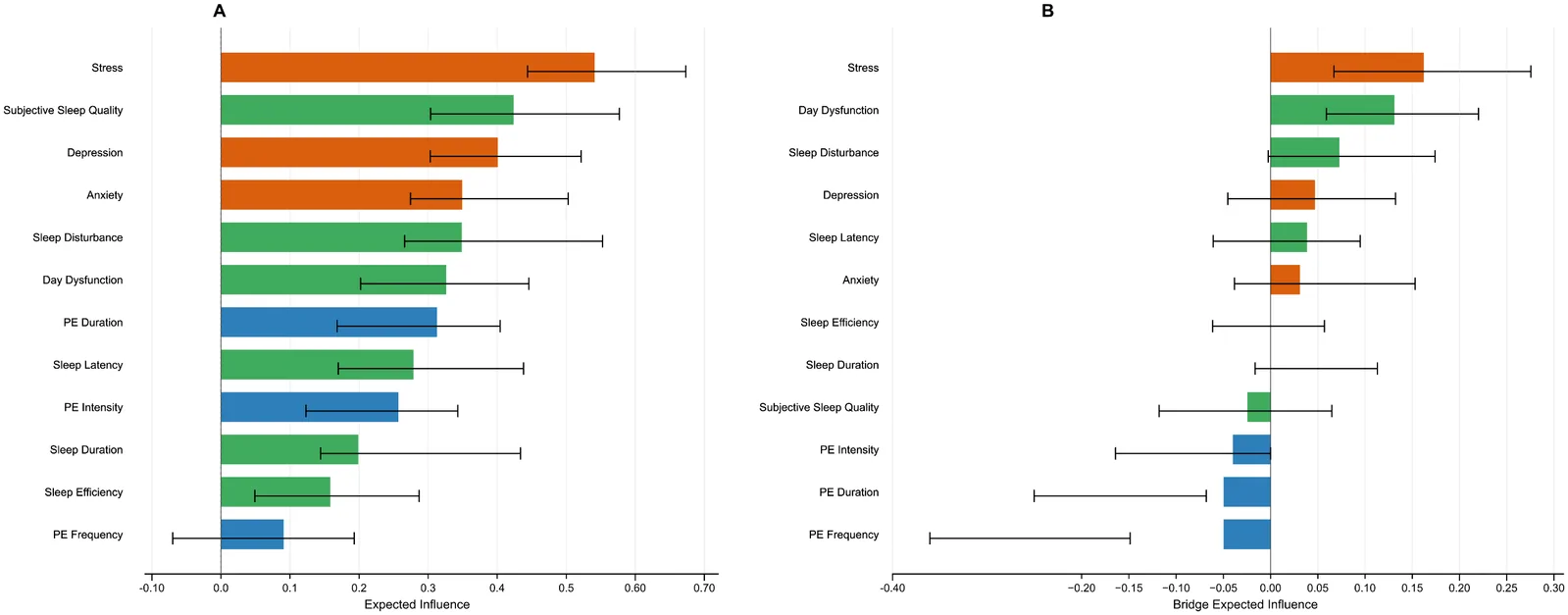
Expected influence and bridge expected influence in the full-sample network. Note. Panel A presents expected influence (EI), and Panel B presents bridge expected influence (BEI). Bars represent the original point estimates, and error bars represent 95% percentile intervals from the nonparametric bootstrap. BEI retains the signs of cross-community edges; negative values indicate predominantly negative cross-community associations and should not be interpreted as weaker bridge effects, causal effects, or protective effects.

The bridge expected influence results showed that stress had the highest positive BEI point estimate (BEI = 0.162), followed by daytime dysfunction (BEI = 0.131) and sleep disturbances (BEI = 0.073). Bootstrap difference tests indicated that the BEI of stress was not clearly distinguishable from those of daytime dysfunction, sleep disturbances, depression, or anxiety (S8 Table). Exercise frequency showed the largest magnitude of negative BEI (BEI = −0.215), followed by exercise duration (BEI = −0.113), but their difference was also not clearly distinguishable. Stress had the largest absolute bridge strength point estimate (0.387), followed by exercise frequency (0.215), subjective sleep quality (0.211), and daytime dysfunction (0.192) (Table 3). BEI preserves the sign of edge weights, and its positive or negative values reflect the overall direction of cross-community connections. It cannot be used to infer causal or temporal direction, nor can negative BEI values be directly interpreted as protective effects.

### Network accuracy and stability

The results of 1,000 nonparametric bootstrap iterations showed that the confidence intervals of stronger edges were generally relatively narrow, whereas the confidence intervals of some weaker edges were wider, suggesting that fine-grained ranking of smaller edge weights should be interpreted with caution. Among the 27 non-zero edges, 16 edges had 95% confidence intervals that did not include zero. The bootstrap confidence intervals for all edges are presented in Fig 3. The five cross-domain edges emphasized in the main text were retained in 99.9%–100.0% of successful bootstrap samples, indicating high retention stability (S9 Table). These bootstrap intervals and retention frequencies reflect sampling variability and edge-selection stability and should not be interpreted as conventional significance tests.

**Fig 3:**
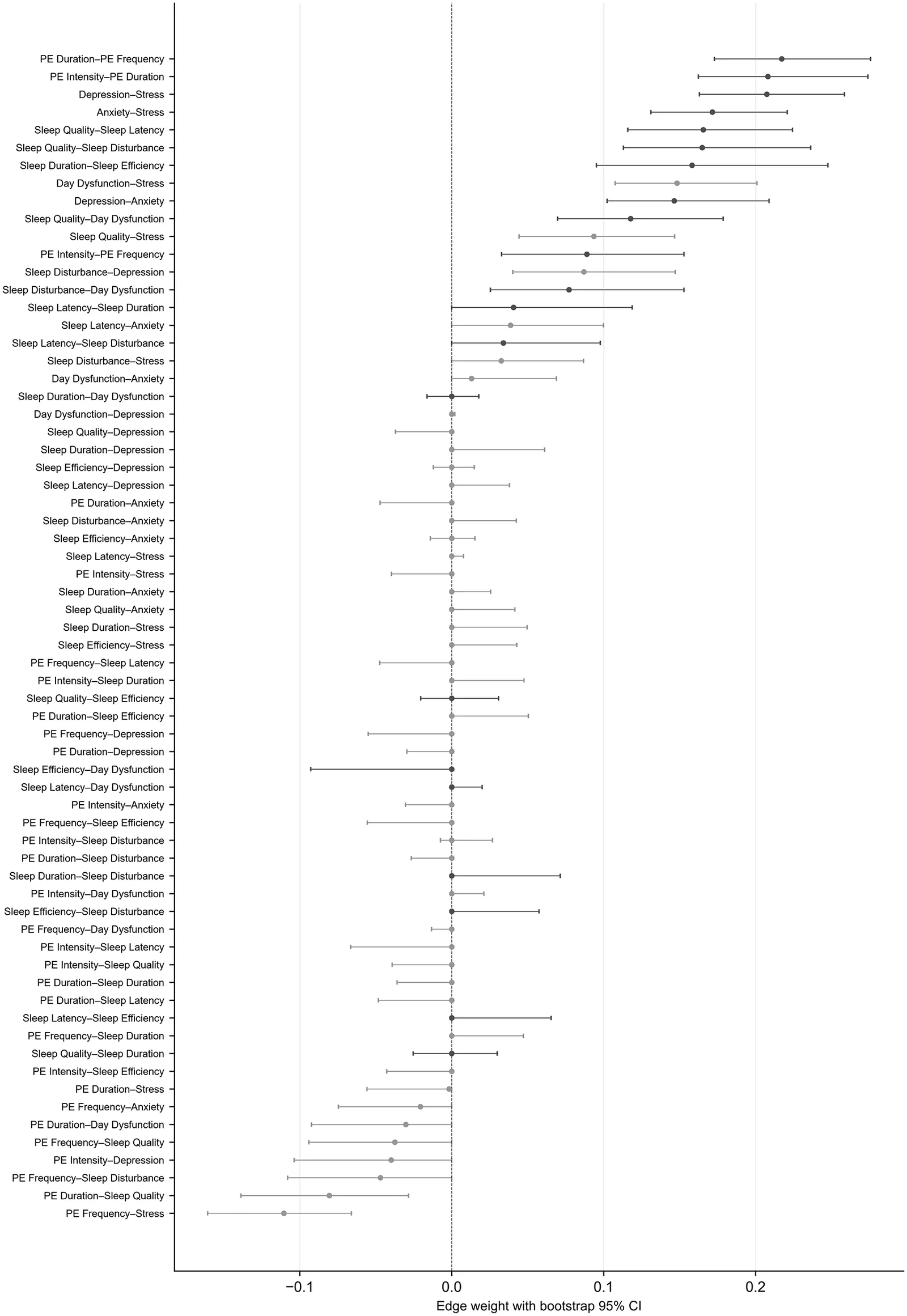
Edge-weight nonparametric bootstrap 95% confidence intervals. Note. Points represent estimated edge weights, and horizontal lines represent 95% percentile intervals from the nonparametric bootstrap. The vertical reference line at zero indicates no association.

The case-dropping bootstrap results showed that the correlation stability coefficients (CS; cor = 0.70) for EI and BEI were 0.60 and 0.65, respectively, both indicating acceptable stability (see Fig 4).

**Fig 4:**
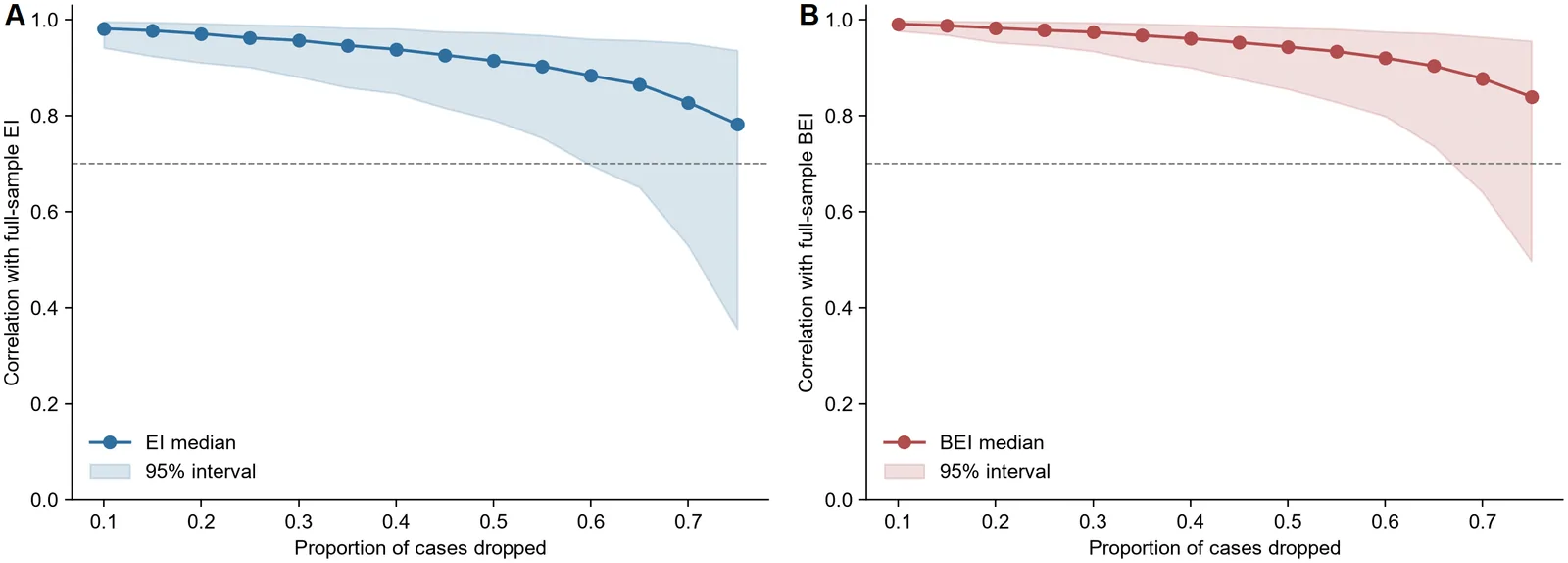
Case-dropping bootstrap stability of expected influence and bridge expected influence. Note. Panel A presents the case-dropping bootstrap stability of expected influence (EI), and Panel B presents the case-dropping bootstrap stability of bridge expected influence (BEI). Solid points and lines represent the median correlation with the full-sample estimates, shaded areas represent the 95% intervals, and the dashed horizontal line indicates a correlation of 0.70.

### Comparison of male and female networks

The global strengths of the male and female networks were 2.692 and 2.191, respectively. The Network Comparison Test showed that the difference in global strength between the two groups was not statistically significant (S = 0.501, P = 0.381), and the difference in overall network structure was also not statistically significant (M = 0.091, P = 0.911). In the comparison of 66 specific edges, no significant gender-differential edges were identified after Bonferroni correction and Benjamini–Hochberg false discovery rate correction. The male and female networks retained 30 and 25 non-zero edges, respectively. Among the 10 largest edge-weight differences, eight showed larger absolute edge weights in males, although none remained significant after multiple-comparison correction. Sex-specific case-dropping analyses showed EI CS coefficients of 0.283 and 0.361 and BEI CS coefficients of 0.361 and 0.516 for males and females, respectively, indicating limited stability for several subgroup centrality estimates. When the female sample was randomly matched to the male sample size (n = 674 per group), no differences were detected in global strength (S = 0.698, P = 0.224) or network structure (M = 0.096, P = 0.891). Across 100 repeated matched subsamples, P < 0.05 occurred in 1% of global-strength tests and none of the network-structure tests. The observed-effect simulation yielded detection proportions of 6% for global strength and 18% for network structure and was therefore treated as exploratory evidence of limited detection capability (S10 Table). Taken together, no gender-related network differences were detected under the current sample and analytical framework; these findings should not be interpreted as evidence that the male and female networks are equivalent or identical (see Fig 1, Table 4, S3 and S10 Tables).

**Table 4:** Network comparison test results for global strength, network structure, and the 10 largest edge-level differences between male and female networks.

Section	Edge	Male global strength	Female global strength	S	Global-strength p	M	Network-structure p	Male weight	Female weight	Absolute difference	Edge-difference p	Bonferroni-adjusted p	BH-FDR q
Overall tests	Global strength	2.692	2.191	0.501	0.381								
Overall tests	Network structure					0.091	0.911						
Edge-level tests	Sleep Disturbance–Day Dysfunction							0.123	0.032	0.091	0.160	1.000	1.000
Edge-level tests	PE Intensity–Depression							−0.079	0.000	0.079	0.106	1.000	1.000
Edge-level tests	PE Intensity–PE Duration							0.240	0.163	0.078	0.205	1.000	1.000
Edge-level tests	Sleep Disturbance–Stress							0.065	0.001	0.064	0.173	1.000	1.000
Edge-level tests	Sleep Latency–Sleep Disturbance							0.060	0.000	0.060	0.315	1.000	1.000
Edge-level tests	Sleep Latency–Anxiety							0.055	0.000	0.055	0.355	1.000	1.000
Edge-level tests	PE Intensity–PE Frequency							0.053	0.101	0.047	0.453	1.000	1.000
Edge-level tests	Sleep Efficiency–Sleep Disturbance							−0.013	0.031	0.044	0.028	1.000	0.615
Edge-level tests	Sleep Duration–Sleep Efficiency							0.165	0.126	0.039	0.656	1.000	1.000
Edge-level tests	Depression–Anxiety							0.155	0.121	0.033	0.603	1.000	1.000

Note. S represents the difference in global strength; M represents the maximum difference in edge weights between the two networks. Absolute difference represents the absolute difference between male and female edge weights. BH-FDR = Benjamini–Hochberg false discovery rate.

### Sensitivity analysis

After adjusting the EBIC hyperparameter from γ = 0.50 to γ = 1.00, both models retained 27 non-zero edges, with no additional edges added or existing edges removed. The Pearson correlation, Spearman correlation, and Jaccard index of non-zero edges between the two models were all 1.000. The maximum absolute difference across all 66 edge weights was 0.0000, confirming that the two estimated edge-weight matrices were identical. The rank correlations of EI and BEI were also both 1.000. The stress symptom node remained the node with the highest EI point estimate and the highest positive BEI point estimate (see S1 Fig).

After incorporating the use of sleep medication into the network, the resulting 13-node model retained 32 non-zero edges. The proportion of zero values for the use of sleep medication node was 97.0%. The stress symptom node maintained the first EI rank in both the primary network and the 13-node network. The rank correlations of EI and BEI among common nodes were 0.972 and 0.923, respectively. The centrality results of the 13-node model are presented in S4 Table.

After re-estimating the network using the Spearman correlation matrix, the Pearson correlation of edge weights was 0.984, the Spearman correlation of edge weights was 0.979, and the rank correlations of EI and BEI were 0.972 and 0.963, respectively, indicating that the main analysis results were generally robust.

After residualizing the 12 nodes for institution, grade, and age, the adjusted network retained 30 non-zero edges compared with 27 in the primary network. All 27 edges retained in the primary network remained non-zero after adjustment, yielding a Jaccard index of 0.900. The Pearson and Spearman correlations of all 66 edge weights between the two networks were 0.990 and 0.972, respectively, while the rank correlations for EI and BEI were 0.979 and 0.977. The five main cross-domain edges retained both their directions and non-zero status after adjustment, indicating that the principal network pattern was generally preserved (S5 Table).

No node pair met the prespecified redundancy threshold (wTO ≥ 0.25; maximum = 0.214). EGA identified four communities: the physical exercise and psychological distress nodes each formed intact communities, whereas the PSQI components separated into two communities, with sleep latency unassigned; four communities were recovered in 71.6% of bootstrap samples (S6 Table). Recalculation of BEI using the empirical communities showed moderate concordance with the prespecified three-community solution (Pearson r = 0.801; Spearman ρ = 0.750). Exercise frequency remained the largest-magnitude negative BEI node, while stress and daytime dysfunction retained relatively high positive BEI values; several PSQI bridge estimates changed, indicating partial sensitivity to community specification (S7 Table).

## Discussion

This study employed a network analysis approach to examine the conditional associations among different dimensions of physical exercise, sleep quality, and psychological distress in university students. The results showed that stronger connections were primarily concentrated within the three domains, whereas cross-domain connections were relatively weaker and concentrated on a limited number of specific nodes. However, the stronger within-domain connections should be interpreted cautiously because nodes within each domain were derived from the same instrument and may share measurement characteristics. The stress symptom node had the highest EI point estimate and the highest positive BEI point estimate; however, its EI was not clearly distinguishable from those of subjective sleep quality and depression, and its BEI was not clearly distinguishable from those of daytime dysfunction, sleep disturbances, depression, or anxiety. Daytime dysfunction–stress showed the largest positive cross-domain point estimate, whereas exercise frequency–stress symptoms and exercise duration–subjective sleep quality were relatively prominent negative connections. In addition, no gender-related network differences were detected under the current sample and analytical framework. Overall, physical exercise, sleep quality, and psychological distress were not universally closely interconnected across all dimensions. Their cross-domain associations were primarily concentrated in nodes such as stress symptoms, daytime dysfunction, exercise frequency, and exercise duration. Bridge estimates for several PSQI components also varied with empirical community specification, indicating that BEI rankings are partly conditional on how communities are defined.

Stress symptoms represented a relatively prominent psychological distress node in terms of associations in this study. Although it had the highest EI point estimate, its centrality differences from subjective sleep quality and depression were not clearly distinguished. Similarly, although stress had the highest positive BEI point estimate and the largest absolute bridge strength point estimate, its signed BEI was not clearly distinguishable from several other nodes. Therefore, it should not be considered the only or definitive core node in the network. A systematic review and meta-analysis of undergraduate students showed that stress was consistently associated with poor sleep quality and insomnia symptoms at a moderate level [32]. A network study of university students with different stress levels also found that the associations among depression, anxiety, and sleep symptoms became more complex as stress levels increased [33]. Furthermore, longitudinal studies suggest that sleep and stress may have a bidirectional relationship: stress can disrupt sleep, whereas persistently poor sleep may also reduce individuals’ ability to cope with daily demands and regulate emotions [34,35].

Daytime dysfunction–stress was one of the more prominent positive cross-domain connections in this study. After controlling for other network nodes, more severe daytime functional impairment was positively associated with higher stress symptom scores. Daytime dysfunction primarily reflects sleep-related daytime sleepiness, reduced energy, and difficulties in maintaining daily activities. A previous study among university students found that daytime dysfunction was associated with depressive symptoms [36]. The effects of sleep problems on university students may not only manifest as insufficient nighttime sleep but may also be reflected in daytime sleepiness, reduced energy, impaired attention, and decreased academic efficiency [37,38]. Therefore, compared with focusing solely on sleep duration, daytime functional status may be more helpful for identifying students who are simultaneously affected by sleep problems and psychological distress.

This study also found a negative connection between exercise frequency and stress, indicating that, after controlling for other physical exercise, sleep, and psychological distress nodes, students who exercised more frequently generally reported lower levels of stress. This pattern is consistent with previous longitudinal and observational evidence linking greater physical activity with lower stress and better recovery among university students [39,40]. However, the present analysis did not assess the psychological or physiological processes underlying this association. Furthermore, systematic reviews and meta-analyses of physical activity interventions have indicated that physical activity may reduce stress, anxiety, and depressive symptoms among university students. However, the heterogeneity and risk of bias in existing studies are relatively high; therefore, the effects require further confirmation through more rigorously designed intervention studies [41].

The negative connection between exercise duration and subjective sleep quality scores indicates that students with longer exercise durations generally report better subjective sleep quality. This pattern is broadly consistent with previous research on physical activity and sleep among university students, although existing evidence remains heterogeneous. A systematic review of physical activity and sleep among university students showed that most studies used cross-sectional designs. Definitions of physical activity intensity, frequency, and sleep indicators varied substantially across studies, and the overall association between physical activity and sleep quality was relatively weak with considerable heterogeneity [13].

It is noteworthy that exercise frequency and duration, rather than exercise intensity, showed more prominent cross-domain connections with sleep and psychological distress. However, this pattern should not be interpreted as evidence that frequency or duration is inherently more important than intensity. The three exercise dimensions were interrelated, and differences in their response distributions may also have influenced their estimated network connections. In addition, the PARS-3 did not assess actual energy expenditure, exercise type, context, or timing. Therefore, the relative prominence of frequency and duration should be regarded as a descriptive feature of the present network rather than a ranking of the importance of different exercise dimensions.

No statistically significant gender-related differences were detected in overall network structure, global strength, or corrected specific edges. Although Yang et al. (2025) and Feng et al. (2026) reported gender-related differences in physical exercise–sleep networks [20,21], the present non-detection should not be interpreted as evidence that these previous findings are inconsistent. Sex-specific centrality estimates showed limited stability for several indices, and the exploratory simulation suggested limited detection capability for global-strength and network-structure differences. The matched-sample analyses yielded a similar non-detection pattern, suggesting that unequal group sizes alone did not account for the result. Differences across studies may also reflect sleep measures, included network nodes, sample characteristics, network-estimation parameters, and multiple-comparison procedures. Therefore, the present findings indicate only that gender-related network differences were not detected under the current sample and analytical framework, rather than demonstrating equivalence between the male and female networks.

The above findings may help identify specific dimensions that warrant further investigation in university health research. In the present network, stress symptoms, daytime dysfunction, exercise frequency, and exercise duration showed relatively prominent cross-domain associations. These findings do not establish that these dimensions should be prioritized as intervention targets, but they may inform the selection of variables for subsequent longitudinal and intervention studies. Future studies could examine whether changes in stress symptoms, daytime functioning, and exercise participation precede changes in other domains and whether these associations can be replicated across different university populations. Because centrality and bridge centrality are descriptive network indices, the intervention relevance of these dimensions should be evaluated directly in prospective and experimental studies.

The strengths of this study include the simultaneous retention of specific dimensions of physical exercise and sleep, which enabled the identification of specific connections such as daytime dysfunction–stress symptoms, exercise frequency–stress symptoms, and exercise duration–subjective sleep quality. Centrality estimates in the full-sample network demonstrated acceptable stability, and the main findings were generally consistent across different parameter settings and node processing approaches. However, this study has several limitations. First, cross-sectional networks cannot determine the temporal order or causal direction among variables. Second, physical exercise, sleep, and psychological distress were all assessed using self-report instruments. In addition, measurement precision differed across nodes. The three PARS-3 nodes represented single-item, five-category self-reported facets of physical exercise and did not capture exercise type, context, or timing. Therefore, measurement precision was not uniform across the network, and lower measurement precision in some nodes may have influenced estimated edge weights and centrality indices. Internal-consistency reliability cannot be estimated for single-item nodes. The PSQI component scores and DASS-21 subscale scores were also based on different item compositions and measurement structures, so their measurement precision may not have been equivalent across nodes. Moreover, because nodes within each domain were derived from the same instrument, stronger within-domain connections may partly reflect shared item content, response format, and recall period rather than only substantive associations among the constructs. Although the redundancy analysis did not identify any node pair exceeding the prespecified topological-overlap threshold, partial overlap in item content or measurement characteristics may still have contributed to some within-domain connections. The recall periods of different scales were not entirely consistent, which may have introduced measurement bias. Third, this study used convenience sampling, and participants were recruited only from first- and second-year students at two institutions in central China. The network was not replicated in an independent sample; therefore, the reproducibility of specific edge weights, centrality patterns, and bridge estimates across universities, regions, or cultural contexts remains uncertain. The male and female groups were unequal in size. Although matched-sample sensitivity analyses produced a similar pattern of non-detection, several sex-specific centrality estimates showed limited stability and the exploratory simulation indicated limited capability to detect gender-related network differences; therefore, the NCT results should not be interpreted as evidence of network equivalence. In addition, depression, anxiety, and stress were represented as nodes using DASS-21 subscale scores. The observed correlations among the three subscales were relatively modest in this sample. Although score verification and the three-factor CFA supported the use of the three subscales as distinct dimensions, their comparatively weak intercorrelations may have reduced the magnitude of within-domain connections among the psychological distress nodes and should be considered when interpreting their network connectivity. This approach facilitates the presentation of overall relationships among the three psychological distress dimensions but cannot reveal heterogeneity among specific symptom items. The primary network was estimated without covariate adjustment. Although a sensitivity analysis adjusting for institution, grade, and age showed that the principal network pattern was largely preserved, potentially relevant factors such as academic stress, financial stress, screen use, caffeine intake, social support, and chronotype were not measured. Residual confounding by these unmeasured factors may therefore have influenced some cross-domain edges. In particular, the DASS-21 stress node cannot be used to infer that any specific type of life stress was responsible for the observed network connections. Finally, the network analysis was exploratory and was not preregistered. Although the robustness of the findings was examined through bootstrap procedures and multiple sensitivity analyses, data-driven features such as the relative prominence of individual edges and bridge nodes should be considered hypothesis-generating and require confirmation in independent, preregistered studies.

## Conclusion

This study found that stronger connections among physical exercise, sleep quality, and psychological distress in university students were primarily concentrated within their respective domains, whereas cross-domain associations were concentrated on a limited number of specific nodes. Stress showed relatively high centrality and cross-community connectivity, while daytime dysfunction–stress was among the more prominent sleep–psychological distress connections. Exercise frequency and duration were respectively associated with lower stress levels and fewer subjective sleep problems. No gender-related network differences were detected under the current sample and analytical framework, although this should not be interpreted as evidence that the male and female networks are equivalent. These findings provide evidence for understanding the dimension-specific associations among physical exercise, sleep quality, and psychological distress in university students. However, their temporal order and intervention value require further validation through longitudinal studies and randomized controlled trials.

## Supporting information

S1 Table All 66 primary-network edges. (DOCX)

S2 Table Nonzero edges with bootstrap confidence intervals. (DOCX)

S3 Table All NCT edge-level differences. (DOCX)

S4 Table Sleep Medication 13-node sensitivity model. (DOCX)

S5 Table Comparison of the primary network and the covariate-adjusted residualized network controlling for institution type, grade, and age. (DOCX)

S6 Table Node redundancy and empirical community assessment using UVA and EGA. (DOCX)

S7 Table Sensitivity of bridge indices to the EGA-derived empirical community structure. (DOCX)

S8 Table Bootstrap difference tests for bridge expected influence. (DOCX)

S9 Table Edge retention frequencies and bootstrap difference tests for cross-domain edges. (DOCX)

S10 Table Gender-network comparison, stability, matched-sample sensitivity, and simulation-based evaluation. (DOCX)

S11 Table Global PSQI score and DASS-21 severity distributions in the study sample. (DOCX)

S1 Fig Gamma = 0.50 versus gamma = 1.00 edge-weight comparison. (DOCX)
