# Supplementary material for: Network analysis of physical exercise, sleep quality, and psychological distress among Chinese university students
Source: PLoS One. 2026 Nov 12;21(11):e0362609. doi: 10.1371/journal.pone.0362609 (PMC13759045; doi:10.1371/journal.pone.0362609)
Supplement: Supplementary file 1 [file pone.0362609.s001.docx]

## Table S1. All 66 primary-network edges

| **Edge ID** | **Node 1** | **Node 2** | **Edge weight** | **Absolute edge weight** | **Bootstrap 95% CI low** | **Bootstrap 95% CI high** | **Community 1** | **Community 2** | **Edge type** |
| --- | --- | --- | --- | --- | --- | --- | --- | --- | --- |
| 1.0000 | PE Intensity | PE Duration | 0.2080 | 0.2080 | 0.1624 | 0.2740 | PE | PE | within-domain |
| 2.0000 | PE Intensity | PE Frequency | 0.0889 | 0.0889 | 0.0328 | 0.1529 | PE | PE | within-domain |
| 3.0000 | PE Duration | PE Frequency | 0.2172 | 0.2172 | 0.1729 | 0.2757 | PE | PE | within-domain |
| 4.0000 | PE Intensity | Sleep Quality | 0.0000 | 0.0000 | -0.0391 | 0.0000 | PE | PSQI | cross-domain |
| 5.0000 | PE Duration | Sleep Quality | -0.0805 | 0.0805 | -0.1387 | -0.0283 | PE | PSQI | cross-domain |
| 6.0000 | PE Frequency | Sleep Quality | -0.0374 | 0.0374 | -0.0940 | 0.0000 | PE | PSQI | cross-domain |
| 7.0000 | PE Intensity | Sleep Latency | 0.0000 | 0.0000 | -0.0665 | 0.0000 | PE | PSQI | cross-domain |
| 8.0000 | PE Duration | Sleep Latency | 0.0000 | 0.0000 | -0.0483 | 0.0000 | PE | PSQI | cross-domain |
| 9.0000 | PE Frequency | Sleep Latency | 0.0000 | 0.0000 | -0.0473 | 0.0000 | PE | PSQI | cross-domain |
| 10.0000 | Sleep Quality | Sleep Latency | 0.1655 | 0.1655 | 0.1159 | 0.2243 | PSQI | PSQI | within-domain |
| 11.0000 | PE Intensity | Sleep Duration | 0.0000 | 0.0000 | 0.0000 | 0.0476 | PE | PSQI | cross-domain |
| 12.0000 | PE Duration | Sleep Duration | 0.0000 | 0.0000 | -0.0360 | 0.0000 | PE | PSQI | cross-domain |
| 13.0000 | PE Frequency | Sleep Duration | 0.0000 | 0.0000 | 0.0000 | 0.0472 | PE | PSQI | cross-domain |
| 14.0000 | Sleep Quality | Sleep Duration | 0.0000 | 0.0000 | -0.0253 | 0.0299 | PSQI | PSQI | within-domain |
| 15.0000 | Sleep Latency | Sleep Duration | 0.0406 | 0.0406 | 0.0000 | 0.1188 | PSQI | PSQI | within-domain |
| 16.0000 | PE Intensity | Sleep Efficiency | 0.0000 | 0.0000 | -0.0428 | 0.0000 | PE | PSQI | cross-domain |
| 17.0000 | PE Duration | Sleep Efficiency | 0.0000 | 0.0000 | 0.0000 | 0.0504 | PE | PSQI | cross-domain |
| 18.0000 | PE Frequency | Sleep Efficiency | 0.0000 | 0.0000 | -0.0555 | 0.0000 | PE | PSQI | cross-domain |
| 19.0000 | Sleep Quality | Sleep Efficiency | 0.0000 | 0.0000 | -0.0204 | 0.0308 | PSQI | PSQI | within-domain |
| 20.0000 | Sleep Latency | Sleep Efficiency | 0.0000 | 0.0000 | 0.0000 | 0.0654 | PSQI | PSQI | within-domain |
| 21.0000 | Sleep Duration | Sleep Efficiency | 0.1582 | 0.1582 | 0.0952 | 0.2476 | PSQI | PSQI | within-domain |
| 22.0000 | PE Intensity | Sleep Disturbance | 0.0000 | 0.0000 | -0.0074 | 0.0268 | PE | PSQI | cross-domain |
| 23.0000 | PE Duration | Sleep Disturbance | 0.0000 | 0.0000 | -0.0266 | 0.0000 | PE | PSQI | cross-domain |
| 24.0000 | PE Frequency | Sleep Disturbance | -0.0468 | 0.0468 | -0.1080 | 0.0000 | PE | PSQI | cross-domain |
| 25.0000 | Sleep Quality | Sleep Disturbance | 0.1648 | 0.1648 | 0.1130 | 0.2363 | PSQI | PSQI | within-domain |
| 26.0000 | Sleep Latency | Sleep Disturbance | 0.0340 | 0.0340 | 0.0000 | 0.0978 | PSQI | PSQI | within-domain |
| 27.0000 | Sleep Duration | Sleep Disturbance | 0.0000 | 0.0000 | 0.0000 | 0.0714 | PSQI | PSQI | within-domain |
| 28.0000 | Sleep Efficiency | Sleep Disturbance | 0.0000 | 0.0000 | 0.0000 | 0.0575 | PSQI | PSQI | within-domain |
| 29.0000 | PE Intensity | Day Dysfunction | 0.0000 | 0.0000 | 0.0000 | 0.0212 | PE | PSQI | cross-domain |
| 30.0000 | PE Duration | Day Dysfunction | -0.0302 | 0.0302 | -0.0923 | 0.0000 | PE | PSQI | cross-domain |
| 31.0000 | PE Frequency | Day Dysfunction | 0.0000 | 0.0000 | -0.0132 | 0.0000 | PE | PSQI | cross-domain |
| 32.0000 | Sleep Quality | Day Dysfunction | 0.1178 | 0.1178 | 0.0697 | 0.1787 | PSQI | PSQI | within-domain |
| 33.0000 | Sleep Latency | Day Dysfunction | 0.0000 | 0.0000 | 0.0000 | 0.0201 | PSQI | PSQI | within-domain |
| 34.0000 | Sleep Duration | Day Dysfunction | 0.0000 | 0.0000 | -0.0163 | 0.0179 | PSQI | PSQI | within-domain |
| 35.0000 | Sleep Efficiency | Day Dysfunction | 0.0000 | 0.0000 | -0.0927 | 0.0000 | PSQI | PSQI | within-domain |
| 36.0000 | Sleep Disturbance | Day Dysfunction | 0.0772 | 0.0772 | 0.0254 | 0.1529 | PSQI | PSQI | within-domain |
| 37.0000 | PE Intensity | Depression | -0.0399 | 0.0399 | -0.1036 | 0.0000 | PE | DASS | cross-domain |
| 38.0000 | PE Duration | Depression | 0.0000 | 0.0000 | -0.0295 | 0.0000 | PE | DASS | cross-domain |
| 39.0000 | PE Frequency | Depression | 0.0000 | 0.0000 | -0.0549 | 0.0000 | PE | DASS | cross-domain |
| 40.0000 | Sleep Quality | Depression | 0.0000 | 0.0000 | -0.0370 | 0.0000 | PSQI | DASS | cross-domain |
| 41.0000 | Sleep Latency | Depression | 0.0000 | 0.0000 | 0.0000 | 0.0380 | PSQI | DASS | cross-domain |
| 42.0000 | Sleep Duration | Depression | 0.0000 | 0.0000 | 0.0000 | 0.0612 | PSQI | DASS | cross-domain |
| 43.0000 | Sleep Efficiency | Depression | 0.0000 | 0.0000 | -0.0121 | 0.0148 | PSQI | DASS | cross-domain |
| 44.0000 | Sleep Disturbance | Depression | 0.0870 | 0.0870 | 0.0402 | 0.1472 | PSQI | DASS | cross-domain |
| 45.0000 | Day Dysfunction | Depression | 0.0000 | 0.0000 | 0.0000 | 0.0021 | PSQI | DASS | cross-domain |
| 46.0000 | PE Intensity | Anxiety | 0.0000 | 0.0000 | -0.0304 | 0.0000 | PE | DASS | cross-domain |
| 47.0000 | PE Duration | Anxiety | 0.0000 | 0.0000 | -0.0472 | 0.0000 | PE | DASS | cross-domain |
| 48.0000 | PE Frequency | Anxiety | -0.0206 | 0.0206 | -0.0746 | 0.0000 | PE | DASS | cross-domain |
| 49.0000 | Sleep Quality | Anxiety | 0.0000 | 0.0000 | 0.0000 | 0.0416 | PSQI | DASS | cross-domain |
| 50.0000 | Sleep Latency | Anxiety | 0.0387 | 0.0387 | 0.0000 | 0.0998 | PSQI | DASS | cross-domain |
| 51.0000 | Sleep Duration | Anxiety | 0.0000 | 0.0000 | 0.0000 | 0.0256 | PSQI | DASS | cross-domain |
| 52.0000 | Sleep Efficiency | Anxiety | 0.0000 | 0.0000 | -0.0140 | 0.0153 | PSQI | DASS | cross-domain |
| 53.0000 | Sleep Disturbance | Anxiety | 0.0000 | 0.0000 | 0.0000 | 0.0425 | PSQI | DASS | cross-domain |
| 54.0000 | Day Dysfunction | Anxiety | 0.0130 | 0.0130 | 0.0000 | 0.0688 | PSQI | DASS | cross-domain |
| 55.0000 | Depression | Anxiety | 0.1464 | 0.1464 | 0.1023 | 0.2089 | DASS | DASS | within-domain |
| 56.0000 | PE Intensity | Stress | 0.0000 | 0.0000 | -0.0396 | 0.0000 | PE | DASS | cross-domain |
| 57.0000 | PE Duration | Stress | -0.0018 | 0.0018 | -0.0557 | 0.0000 | PE | DASS | cross-domain |
| 58.0000 | PE Frequency | Stress | -0.1105 | 0.1105 | -0.1607 | -0.0660 | PE | DASS | cross-domain |
| 59.0000 | Sleep Quality | Stress | 0.0935 | 0.0935 | 0.0443 | 0.1468 | PSQI | DASS | cross-domain |
| 60.0000 | Sleep Latency | Stress | 0.0000 | 0.0000 | 0.0000 | 0.0078 | PSQI | DASS | cross-domain |
| 61.0000 | Sleep Duration | Stress | 0.0000 | 0.0000 | 0.0000 | 0.0497 | PSQI | DASS | cross-domain |
| 62.0000 | Sleep Efficiency | Stress | 0.0000 | 0.0000 | 0.0000 | 0.0429 | PSQI | DASS | cross-domain |
| 63.0000 | Sleep Disturbance | Stress | 0.0326 | 0.0326 | 0.0000 | 0.0867 | PSQI | DASS | cross-domain |
| 64.0000 | Day Dysfunction | Stress | 0.1483 | 0.1483 | 0.1076 | 0.2010 | PSQI | DASS | cross-domain |
| 65.0000 | Depression | Stress | 0.2074 | 0.2074 | 0.1630 | 0.2586 | DASS | DASS | within-domain |
| 66.0000 | Anxiety | Stress | 0.1716 | 0.1716 | 0.1311 | 0.2209 | DASS | DASS | within-domain |
