# Supplementary material for: Network analysis of physical exercise, sleep quality, and psychological distress among Chinese university students
Source: PLoS One. 2026 Nov 12;21(11):e0362609. doi: 10.1371/journal.pone.0362609 (PMC13759045; doi:10.1371/journal.pone.0362609)
Supplement: Supplementary file 2 [file pone.0362609.s002.docx]

## Table S2. Nonzero edges with bootstrap confidence intervals

| **Edge ID** | **Node 1** | **Node 2** | **Edge weight** | **Absolute edge weight** | **Bootstrap 95% CI low** | **Bootstrap 95% CI high** | **Community 1** | **Community 2** | **Edge type** |
| --- | --- | --- | --- | --- | --- | --- | --- | --- | --- |
| 1.0000 | PE Intensity | PE Duration | 0.2080 | 0.2080 | 0.1624 | 0.2740 | PE | PE | within-domain |
| 2.0000 | PE Intensity | PE Frequency | 0.0889 | 0.0889 | 0.0328 | 0.1529 | PE | PE | within-domain |
| 3.0000 | PE Duration | PE Frequency | 0.2172 | 0.2172 | 0.1729 | 0.2757 | PE | PE | within-domain |
| 5.0000 | PE Duration | Sleep Quality | -0.0805 | 0.0805 | -0.1387 | -0.0283 | PE | PSQI | cross-domain |
| 6.0000 | PE Frequency | Sleep Quality | -0.0374 | 0.0374 | -0.0940 | 0.0000 | PE | PSQI | cross-domain |
| 10.0000 | Sleep Quality | Sleep Latency | 0.1655 | 0.1655 | 0.1159 | 0.2243 | PSQI | PSQI | within-domain |
| 15.0000 | Sleep Latency | Sleep Duration | 0.0406 | 0.0406 | 0.0000 | 0.1188 | PSQI | PSQI | within-domain |
| 21.0000 | Sleep Duration | Sleep Efficiency | 0.1582 | 0.1582 | 0.0952 | 0.2476 | PSQI | PSQI | within-domain |
| 24.0000 | PE Frequency | Sleep Disturbance | -0.0468 | 0.0468 | -0.1080 | 0.0000 | PE | PSQI | cross-domain |
| 25.0000 | Sleep Quality | Sleep Disturbance | 0.1648 | 0.1648 | 0.1130 | 0.2363 | PSQI | PSQI | within-domain |
| 26.0000 | Sleep Latency | Sleep Disturbance | 0.0340 | 0.0340 | 0.0000 | 0.0978 | PSQI | PSQI | within-domain |
| 30.0000 | PE Duration | Day Dysfunction | -0.0302 | 0.0302 | -0.0923 | 0.0000 | PE | PSQI | cross-domain |
| 32.0000 | Sleep Quality | Day Dysfunction | 0.1178 | 0.1178 | 0.0697 | 0.1787 | PSQI | PSQI | within-domain |
| 36.0000 | Sleep Disturbance | Day Dysfunction | 0.0772 | 0.0772 | 0.0254 | 0.1529 | PSQI | PSQI | within-domain |
| 37.0000 | PE Intensity | Depression | -0.0399 | 0.0399 | -0.1036 | 0.0000 | PE | DASS | cross-domain |
| 44.0000 | Sleep Disturbance | Depression | 0.0870 | 0.0870 | 0.0402 | 0.1472 | PSQI | DASS | cross-domain |
| 48.0000 | PE Frequency | Anxiety | -0.0206 | 0.0206 | -0.0746 | 0.0000 | PE | DASS | cross-domain |
| 50.0000 | Sleep Latency | Anxiety | 0.0387 | 0.0387 | 0.0000 | 0.0998 | PSQI | DASS | cross-domain |
| 54.0000 | Day Dysfunction | Anxiety | 0.0130 | 0.0130 | 0.0000 | 0.0688 | PSQI | DASS | cross-domain |
| 55.0000 | Depression | Anxiety | 0.1464 | 0.1464 | 0.1023 | 0.2089 | DASS | DASS | within-domain |
| 57.0000 | PE Duration | Stress | -0.0018 | 0.0018 | -0.0557 | 0.0000 | PE | DASS | cross-domain |
| 58.0000 | PE Frequency | Stress | -0.1105 | 0.1105 | -0.1607 | -0.0660 | PE | DASS | cross-domain |
| 59.0000 | Sleep Quality | Stress | 0.0935 | 0.0935 | 0.0443 | 0.1468 | PSQI | DASS | cross-domain |
| 63.0000 | Sleep Disturbance | Stress | 0.0326 | 0.0326 | 0.0000 | 0.0867 | PSQI | DASS | cross-domain |
| 64.0000 | Day Dysfunction | Stress | 0.1483 | 0.1483 | 0.1076 | 0.2010 | PSQI | DASS | cross-domain |
| 65.0000 | Depression | Stress | 0.2074 | 0.2074 | 0.1630 | 0.2586 | DASS | DASS | within-domain |
| 66.0000 | Anxiety | Stress | 0.1716 | 0.1716 | 0.1311 | 0.2209 | DASS | DASS | within-domain |
