# Supplementary material for: Network analysis of physical exercise, sleep quality, and psychological distress among Chinese university students
Source: PLoS One. 2026 Nov 12;21(11):e0362609. doi: 10.1371/journal.pone.0362609 (PMC13759045; doi:10.1371/journal.pone.0362609)
Supplement: Supplementary file 3 [file pone.0362609.s003.docx]

## Table S3. All NCT edge-level differences

| **Node 1** | **Node 2** | **Difference** | **Raw p** | **Bonferroni p** | **BH-FDR q** | **Bonferroni significant** | **BH-FDR significant** |
| --- | --- | --- | --- | --- | --- | --- | --- |
| Sleep Disturbance | Day Dysfunction | 0.0914 | 0.1598 | 1.0000 | 1.0000 | False | False |
| PE Intensity | Depression | 0.0791 | 0.1059 | 1.0000 | 1.0000 | False | False |
| PE Intensity | PE Duration | 0.0777 | 0.2048 | 1.0000 | 1.0000 | False | False |
| Sleep Disturbance | Stress | 0.0642 | 0.1728 | 1.0000 | 1.0000 | False | False |
| Sleep Latency | Sleep Disturbance | 0.0599 | 0.3147 | 1.0000 | 1.0000 | False | False |
| Sleep Latency | Anxiety | 0.0552 | 0.3546 | 1.0000 | 1.0000 | False | False |
| PE Intensity | PE Frequency | 0.0474 | 0.4525 | 1.0000 | 1.0000 | False | False |
| Sleep Efficiency | Sleep Disturbance | 0.0440 | 0.0280 | 1.0000 | 0.6154 | False | False |
| Sleep Duration | Sleep Efficiency | 0.0391 | 0.6563 | 1.0000 | 1.0000 | False | False |
| Depression | Anxiety | 0.0334 | 0.6034 | 1.0000 | 1.0000 | False | False |
| Depression | Stress | 0.0331 | 0.5015 | 1.0000 | 1.0000 | False | False |
| Sleep Disturbance | Depression | 0.0326 | 0.5584 | 1.0000 | 1.0000 | False | False |
| Anxiety | Stress | 0.0321 | 0.5335 | 1.0000 | 1.0000 | False | False |
| PE Duration | Anxiety | 0.0320 | 0.0869 | 1.0000 | 1.0000 | False | False |
| PE Duration | Sleep Quality | 0.0278 | 0.6454 | 1.0000 | 1.0000 | False | False |
| PE Duration | Stress | 0.0277 | 0.1878 | 1.0000 | 1.0000 | False | False |
| Sleep Quality | Sleep Duration | 0.0270 | 0.0160 | 1.0000 | 0.5275 | False | False |
| Day Dysfunction | Anxiety | 0.0258 | 0.5195 | 1.0000 | 1.0000 | False | False |
| PE Frequency | Stress | 0.0238 | 0.6384 | 1.0000 | 1.0000 | False | False |
| Sleep Quality | Stress | 0.0226 | 0.6613 | 1.0000 | 1.0000 | False | False |
| PE Frequency | Anxiety | 0.0208 | 0.5704 | 1.0000 | 1.0000 | False | False |
| Sleep Latency | Sleep Duration | 0.0208 | 0.7842 | 1.0000 | 1.0000 | False | False |
| Sleep Latency | Stress | 0.0131 | 0.0160 | 1.0000 | 0.5275 | False | False |
| Sleep Quality | Day Dysfunction | 0.0109 | 0.8811 | 1.0000 | 1.0000 | False | False |
| PE Frequency | Depression | 0.0084 | 0.5924 | 1.0000 | 1.0000 | False | False |
| PE Frequency | Sleep Duration | 0.0080 | 0.1009 | 1.0000 | 1.0000 | False | False |
| Sleep Quality | Sleep Disturbance | 0.0062 | 0.9231 | 1.0000 | 1.0000 | False | False |
| Day Dysfunction | Stress | 0.0036 | 0.9431 | 1.0000 | 1.0000 | False | False |
| PE Frequency | Sleep Disturbance | 0.0027 | 0.9670 | 1.0000 | 1.0000 | False | False |
| PE Duration | PE Frequency | 0.0020 | 0.9690 | 1.0000 | 1.0000 | False | False |
| PE Duration | Day Dysfunction | 0.0015 | 0.9880 | 1.0000 | 1.0000 | False | False |
| PE Frequency | Sleep Quality | 0.0014 | 0.9760 | 1.0000 | 1.0000 | False | False |
| Sleep Quality | Sleep Latency | 0.0014 | 0.9850 | 1.0000 | 1.0000 | False | False |
| PE Intensity | Sleep Quality | 0.0000 | 1.0000 | 1.0000 | 1.0000 | False | False |
| PE Intensity | Sleep Latency | 0.0000 | 1.0000 | 1.0000 | 1.0000 | False | False |
| PE Duration | Sleep Latency | 0.0000 | 1.0000 | 1.0000 | 1.0000 | False | False |
| PE Frequency | Sleep Latency | 0.0000 | 1.0000 | 1.0000 | 1.0000 | False | False |
| PE Intensity | Sleep Duration | 0.0000 | 1.0000 | 1.0000 | 1.0000 | False | False |
| PE Duration | Sleep Duration | 0.0000 | 1.0000 | 1.0000 | 1.0000 | False | False |
| PE Intensity | Sleep Efficiency | 0.0000 | 1.0000 | 1.0000 | 1.0000 | False | False |
| PE Duration | Sleep Efficiency | 0.0000 | 1.0000 | 1.0000 | 1.0000 | False | False |
| PE Frequency | Sleep Efficiency | 0.0000 | 1.0000 | 1.0000 | 1.0000 | False | False |
| Sleep Quality | Sleep Efficiency | 0.0000 | 1.0000 | 1.0000 | 1.0000 | False | False |
| Sleep Latency | Sleep Efficiency | 0.0000 | 1.0000 | 1.0000 | 1.0000 | False | False |
| PE Intensity | Sleep Disturbance | 0.0000 | 1.0000 | 1.0000 | 1.0000 | False | False |
| PE Duration | Sleep Disturbance | 0.0000 | 1.0000 | 1.0000 | 1.0000 | False | False |
| Sleep Duration | Sleep Disturbance | 0.0000 | 1.0000 | 1.0000 | 1.0000 | False | False |
| PE Intensity | Day Dysfunction | 0.0000 | 1.0000 | 1.0000 | 1.0000 | False | False |
| PE Frequency | Day Dysfunction | 0.0000 | 1.0000 | 1.0000 | 1.0000 | False | False |
| Sleep Latency | Day Dysfunction | 0.0000 | 1.0000 | 1.0000 | 1.0000 | False | False |
| Sleep Duration | Day Dysfunction | 0.0000 | 1.0000 | 1.0000 | 1.0000 | False | False |
| Sleep Efficiency | Day Dysfunction | 0.0000 | 1.0000 | 1.0000 | 1.0000 | False | False |
| PE Duration | Depression | 0.0000 | 1.0000 | 1.0000 | 1.0000 | False | False |
| Sleep Quality | Depression | 0.0000 | 1.0000 | 1.0000 | 1.0000 | False | False |
| Sleep Latency | Depression | 0.0000 | 1.0000 | 1.0000 | 1.0000 | False | False |
| Sleep Duration | Depression | 0.0000 | 1.0000 | 1.0000 | 1.0000 | False | False |
| Sleep Efficiency | Depression | 0.0000 | 1.0000 | 1.0000 | 1.0000 | False | False |
| Day Dysfunction | Depression | 0.0000 | 1.0000 | 1.0000 | 1.0000 | False | False |
| PE Intensity | Anxiety | 0.0000 | 1.0000 | 1.0000 | 1.0000 | False | False |
| Sleep Quality | Anxiety | 0.0000 | 1.0000 | 1.0000 | 1.0000 | False | False |
| Sleep Duration | Anxiety | 0.0000 | 1.0000 | 1.0000 | 1.0000 | False | False |
| Sleep Efficiency | Anxiety | 0.0000 | 1.0000 | 1.0000 | 1.0000 | False | False |
| Sleep Disturbance | Anxiety | 0.0000 | 1.0000 | 1.0000 | 1.0000 | False | False |
| PE Intensity | Stress | 0.0000 | 1.0000 | 1.0000 | 1.0000 | False | False |
| Sleep Duration | Stress | 0.0000 | 1.0000 | 1.0000 | 1.0000 | False | False |
| Sleep Efficiency | Stress | 0.0000 | 1.0000 | 1.0000 | 1.0000 | False | False |
