# Supplementary material for: Network analysis of physical exercise, sleep quality, and psychological distress among Chinese university students
Source: PLoS One. 2026 Nov 12;21(11):e0362609. doi: 10.1371/journal.pone.0362609 (PMC13759045; doi:10.1371/journal.pone.0362609)
Supplement: Supplementary file 4 [file pone.0362609.s004.docx]

## Table S4. Sleep Medication 13-node sensitivity model

| **Section** | **Variable** | **Mean** | **SD** | **Min** | **Max** | **Zero_prop** | **EI** | **EI rank** | **BEI** | **BEI rank** |
| --- | --- | --- | --- | --- | --- | --- | --- | --- | --- | --- |
| Sleep Medication descriptive | Sleep_Medication | 0.0450 | 0.2816 | 0.0000 | 3.0000 | 0.9700 |  |  |  |  |
| 13-node centrality | PE_Intensity |  |  |  |  |  | 0.2769 | 9.0000 | -0.0200 | 9.0000 |
| 13-node centrality | PE_Duration |  |  |  |  |  | 0.3126 | 7.0000 | -0.1126 | 12.0000 |
| 13-node centrality | PE_Frequency |  |  |  |  |  | 0.0908 | 12.0000 | -0.2154 | 13.0000 |
| 13-node centrality | Sleep_Quality |  |  |  |  |  | 0.4237 | 2.0000 | -0.0244 | 10.0000 |
| 13-node centrality | Sleep_Latency |  |  |  |  |  | 0.2960 | 8.0000 | 0.0388 | 4.0000 |
| 13-node centrality | Sleep_Duration |  |  |  |  |  | 0.1985 | 11.0000 | 0.0000 | 6.0000 |
| 13-node centrality | Sleep_Efficiency |  |  |  |  |  | 0.2049 | 10.0000 | 0.0000 | 6.0000 |
| 13-node centrality | Sleep_Disturbance |  |  |  |  |  | 0.3488 | 4.0000 | 0.0727 | 3.0000 |
| 13-node centrality | Day_Dysfunction |  |  |  |  |  | 0.3261 | 6.0000 | 0.1311 | 2.0000 |
| 13-node centrality | Depression |  |  |  |  |  | 0.3425 | 5.0000 | -0.0116 | 8.0000 |
| 13-node centrality | Anxiety |  |  |  |  |  | 0.3490 | 3.0000 | 0.0313 | 5.0000 |
| 13-node centrality | Stress |  |  |  |  |  | 0.5566 | 1.0000 | 0.1771 | 1.0000 |
| 13-node centrality | Sleep_Medication |  |  |  |  |  | 0.0379 | 13.0000 | -0.0260 | 11.0000 |
