# Supplementary material for: Network analysis of physical exercise, sleep quality, and psychological distress among Chinese university students
Source: PLoS One. 2026 Nov 12;21(11):e0362609. doi: 10.1371/journal.pone.0362609 (PMC13759045; doi:10.1371/journal.pone.0362609)
Supplement: Supplementary file 5 [file pone.0362609.s005.docx]

**Table S5. Comparison of the primary network and the covariate-adjusted residualized network controlling for institution type, grade, and age**

| **Category** | **Metric / edge** | **Primary network** | **Adjusted network** | **Comparison** |
| --- | --- | --- | --- | --- |
| Network structure and similarity | Analytic sample size | 1,600 | 1,600 | No change |
| Network structure and similarity | Non-zero edges | 27 | 30 | 27 common; 3 newly retained; 0 lost |
| Network structure and similarity | Network density | 40.9% | 45.5% | +4.6 percentage points |
| Network structure and similarity | Jaccard index | — | — | 0.900 |
| Network structure and similarity | Pearson correlation of all 66 edge weights | — | — | 0.990 |
| Network structure and similarity | Spearman correlation of all 66 edge weights | — | — | 0.972 |
| Network structure and similarity | Mean absolute edge difference | — | — | 0.005 |
| Network structure and similarity | Maximum absolute edge difference | — | — | 0.046 (Sleep Duration–Sleep Efficiency) |
| Centrality and bridge centrality | EI rank correlation | — | — | 0.979 |
| Centrality and bridge centrality | BEI rank correlation | — | — | 0.977 |
| Centrality and bridge centrality | Stress EI | 0.541 | 0.562 | Δ = +0.021 |
| Centrality and bridge centrality | Stress BEI | 0.162 | 0.157 | Δ = −0.005 |
| Key cross-domain edges | Daytime Dysfunction–Stress | 0.148 | 0.150 | \|Δ\| = 0.002; direction retained |
| Key cross-domain edges | Subjective Sleep Quality–Stress | 0.094 | 0.097 | \|Δ\| = 0.003; direction retained |
| Key cross-domain edges | Sleep Disturbance–Depression | 0.087 | 0.082 | \|Δ\| = 0.005; direction retained |
| Key cross-domain edges | PE Frequency–Stress | −0.111 | −0.116 | \|Δ\| = 0.006; direction retained |
| Key cross-domain edges | PE Duration–Subjective Sleep Quality | −0.081 | −0.076 | \|Δ\| = 0.005; direction retained |

**Note.** The primary network was estimated from the 12 original nodes using cor_auto and EBICglasso (γ = 0.50). For the covariate-adjusted sensitivity analysis, each node was residualized for institution type, grade, and age, with institution type and grade treated as categorical variables; the adjusted network was then estimated from the Pearson correlation matrix of the residuals using EBICglasso (γ = 0.50). EI = expected influence; BEI = bridge expected influence; Δ = adjusted minus primary. Non-zero edges indicate edges retained by regularization and are not equivalent to conventional statistical significance.
