# Supplementary material for: Network analysis of physical exercise, sleep quality, and psychological distress among Chinese university students
Source: PLoS One. 2026 Nov 12;21(11):e0362609. doi: 10.1371/journal.pone.0362609 (PMC13759045; doi:10.1371/journal.pone.0362609)
Supplement: Supplementary file 6 [file pone.0362609.s006.docx]

**Table S6. Node redundancy and empirical community assessment using UVA and EGA**

**Panel A. EGA community membership and bootstrap item stability**

| **Node** | **Theoretical community** | **EGA empirical community** | **Item stability** |
| --- | --- | --- | --- |
| PE Intensity | PE | C1 | 1.000 |
| PE Duration | PE | C1 | 1.000 |
| PE Frequency | PE | C1 | 0.998 |
| Subjective Sleep Quality | PSQI | C2 | 0.994 |
| Sleep Latency | PSQI | Unassigned (NA) | NA |
| Sleep Duration | PSQI | C3 | 0.976 |
| Sleep Efficiency | PSQI | C3 | 0.976 |
| Sleep Disturbance | PSQI | C2 | 0.968 |
| Day Dysfunction | PSQI | C2 | 0.908 |
| Depression | DASS | C4 | 0.736 |
| Anxiety | DASS | C4 | 0.736 |
| Stress | DASS | C4 | 0.734 |

**Panel B. Bootstrap structural consistency of the EGA communities**

| **Empirical community** | **Nodes** | **Structural consistency** |
| --- | --- | --- |
| C1 | PE Intensity; PE Duration; PE Frequency | 0.998 |
| C2 | Subjective Sleep Quality; Sleep Disturbance; Day Dysfunction | 0.902 |
| C3 | Sleep Duration; Sleep Efficiency | 0.986 |
| C4 | Depression; Anxiety; Stress | 0.734 |

**Panel C. Bootstrap distribution of the number of communities**

| **Number of communities** | **Proportion of 500 bootstrap samples** |
| --- | --- |
| 3 | 0.284 |
| 4 | 0.716 |

**Note.** UVA = Unique Variable Analysis; EGA = Exploratory Graph Analysis; wTO = weighted topological overlap. UVA used a prespecified redundancy threshold of wTO >= 0.25. No node pair met this threshold. The largest wTO was observed for PE Intensity-PE Duration (0.214); Day Dysfunction-Stress and Sleep Duration-Sleep Efficiency had wTO values of 0.142 and 0.181, respectively. Sleep Latency was unassigned (wc = NA) in the EGA solution; therefore, its item stability was not estimated. Because no node pair met the redundancy threshold, no nodes were combined or deleted.
