# Supplementary material for: Network analysis of physical exercise, sleep quality, and psychological distress among Chinese university students
Source: PLoS One. 2026 Nov 12;21(11):e0362609. doi: 10.1371/journal.pone.0362609 (PMC13759045; doi:10.1371/journal.pone.0362609)
Supplement: Supplementary file 7 [file pone.0362609.s007.docx]

**Table S7. Sensitivity of bridge indices to the EGA-derived empirical community structure**

| **Node** | **Theoretical BEI** | **Empirical-community BEI** | **Delta BEI** | **Theoretical absolute bridge strength** | **Empirical absolute bridge strength** |
| --- | --- | --- | --- | --- | --- |
| PE Intensity | -0.040 | -0.040 | +0.000 | 0.040 | 0.040 |
| PE Duration | -0.113 | -0.113 | +0.000 | 0.113 | 0.113 |
| PE Frequency | -0.215 | -0.215 | +0.000 | 0.215 | 0.215 |
| Subjective Sleep Quality | -0.024 | 0.141 | +0.166 | 0.211 | 0.377 |
| Sleep Latency | 0.039 | 0.279 | +0.240 | 0.039 | 0.279 |
| Sleep Duration | 0.000 | 0.041 | +0.041 | 0.000 | 0.041 |
| Sleep Efficiency | 0.000 | 0.000 | +0.000 | 0.000 | 0.000 |
| Sleep Disturbance | 0.073 | 0.107 | +0.034 | 0.166 | 0.200 |
| Day Dysfunction | 0.131 | 0.131 | +0.000 | 0.192 | 0.192 |
| Depression | 0.047 | 0.047 | +0.000 | 0.127 | 0.127 |
| Anxiety | 0.031 | 0.031 | +0.000 | 0.072 | 0.072 |
| Stress | 0.162 | 0.162 | +0.000 | 0.387 | 0.387 |

**Note.** BEI = bridge expected influence. The primary 12-node, 27-edge network weight matrix was held fixed; only community assignment was changed. The theoretical solution used three communities (physical exercise, PSQI, and DASS). The empirical solution used C1 = PE Intensity/PE Duration/PE Frequency; C2 = Subjective Sleep Quality/Sleep Disturbance/Day Dysfunction; C3 = Sleep Duration/Sleep Efficiency; C4 = Depression/Anxiety/Stress; and Sleep Latency as a singleton community because it was unassigned in the EGA solution. Across the two community specifications, Pearson r for signed BEI = 0.801, Spearman rho for signed BEI = 0.750, Spearman rho for BEI ranks = 0.750, and Spearman rho for absolute bridge strength ranks = 0.764. Treating Sleep Latency as a singleton makes all of its retained edges cross-community edges; therefore, its empirical-community BEI should not be interpreted as evidence that it is intrinsically the most important bridge node.
