# Supplementary material for: Network analysis of physical exercise, sleep quality, and psychological distress among Chinese university students
Source: PLoS One. 2026 Nov 12;21(11):e0362609. doi: 10.1371/journal.pone.0362609 (PMC13759045; doi:10.1371/journal.pone.0362609)
Supplement: Supplementary file 8 [file pone.0362609.s008.docx]

**Table S8. Bootstrap difference tests for bridge expected influence**

| **Node 1** | **Node 2** | **BEI** **Node 1** | **BEI** **Node 2** | **Difference** | **Bootstrap** **mean difference** | **95% CI** **low** | **95% CI** **high** | **CI excludes** **zero** |
| --- | --- | --- | --- | --- | --- | --- | --- | --- |
| PE Intensity | PE Duration | -0.0399 | -0.1125 | 0.0726 | 0.0763 | -0.0542 | 0.1995 | No |
| PE Intensity | PE Frequency | -0.0399 | -0.2154 | 0.1755 | 0.1732 | 0.0364 | 0.3096 | Yes |
| PE Intensity | Subjective Sleep Quality | -0.0399 | -0.0244 | -0.0155 | -0.0477 | -0.1777 | 0.0705 | No |
| PE Intensity | Sleep Latency | -0.0399 | 0.0387 | -0.0786 | -0.0928 | -0.1977 | 0.0024 | No |
| PE Intensity | Sleep Duration | -0.0399 | 0.0000 | -0.0399 | -0.1122 | -0.2427 | -0.0125 | Yes |
| PE Intensity | Sleep Efficiency | -0.0399 | 0.0000 | -0.0399 | -0.0748 | -0.1855 | 0.0147 | No |
| PE Intensity | Sleep Disturbance | -0.0399 | 0.0728 | -0.1127 | -0.1585 | -0.2744 | -0.0482 | Yes |
| PE Intensity | Day Dysfunction | -0.0399 | 0.1311 | -0.1710 | -0.2176 | -0.3408 | -0.1006 | Yes |
| PE Intensity | Depression | -0.0399 | 0.0471 | -0.0870 | -0.1230 | -0.2418 | -0.0223 | Yes |
| PE Intensity | Anxiety | -0.0399 | 0.0311 | -0.0710 | -0.1295 | -0.2647 | -0.0133 | Yes |
| PE Intensity | Stress | -0.0399 | 0.1622 | -0.2021 | -0.2476 | -0.3962 | -0.1192 | Yes |
| PE Duration | PE Frequency | -0.1125 | -0.2154 | 0.1029 | 0.0968 | -0.0513 | 0.2405 | No |
| PE Duration | Subjective Sleep Quality | -0.1125 | -0.0244 | -0.0881 | -0.1241 | -0.2406 | -0.0183 | Yes |
| PE Duration | Sleep Latency | -0.1125 | 0.0387 | -0.1512 | -0.1692 | -0.2805 | -0.0571 | Yes |
| PE Duration | Sleep Duration | -0.1125 | 0.0000 | -0.1125 | -0.1885 | -0.3315 | -0.0745 | Yes |
| PE Duration | Sleep Efficiency | -0.1125 | 0.0000 | -0.1125 | -0.1512 | -0.2566 | -0.0570 | Yes |
| PE Duration | Sleep Disturbance | -0.1125 | 0.0728 | -0.1853 | -0.2349 | -0.3739 | -0.1107 | Yes |
| PE Duration | Day Dysfunction | -0.1125 | 0.1311 | -0.2436 | -0.2940 | -0.4070 | -0.1935 | Yes |
| PE Duration | Depression | -0.1125 | 0.0471 | -0.1596 | -0.1994 | -0.3325 | -0.0684 | Yes |
| PE Duration | Anxiety | -0.1125 | 0.0311 | -0.1436 | -0.2058 | -0.3324 | -0.0814 | Yes |
| PE Duration | Stress | -0.1125 | 0.1622 | -0.2747 | -0.3240 | -0.4728 | -0.1884 | Yes |
| PE Frequency | Subjective Sleep Quality | -0.2154 | -0.0244 | -0.1910 | -0.2209 | -0.3551 | -0.0859 | Yes |
| PE Frequency | Sleep Latency | -0.2154 | 0.0387 | -0.2541 | -0.2660 | -0.3929 | -0.1277 | Yes |
| PE Frequency | Sleep Duration | -0.2154 | 0.0000 | -0.2154 | -0.2854 | -0.4336 | -0.1537 | Yes |
| PE Frequency | Sleep Efficiency | -0.2154 | 0.0000 | -0.2154 | -0.2480 | -0.3670 | -0.1306 | Yes |
| PE Frequency | Sleep Disturbance | -0.2154 | 0.0728 | -0.2882 | -0.3317 | -0.4632 | -0.2087 | Yes |
| PE Frequency | Day Dysfunction | -0.2154 | 0.1311 | -0.3465 | -0.3908 | -0.5250 | -0.2516 | Yes |
| PE Frequency | Depression | -0.2154 | 0.0471 | -0.2625 | -0.2962 | -0.4348 | -0.1550 | Yes |
| PE Frequency | Anxiety | -0.2154 | 0.0311 | -0.2465 | -0.3026 | -0.4367 | -0.1640 | Yes |
| PE Frequency | Stress | -0.2154 | 0.1622 | -0.3776 | -0.4208 | -0.5704 | -0.2846 | Yes |
| Subjective Sleep Quality | Sleep Latency | -0.0244 | 0.0387 | -0.0631 | -0.0451 | -0.1683 | 0.0826 | No |
| Subjective Sleep Quality | Sleep Duration | -0.0244 | 0.0000 | -0.0244 | -0.0644 | -0.2033 | 0.0479 | No |
| Subjective Sleep Quality | Sleep Efficiency | -0.0244 | 0.0000 | -0.0244 | -0.0271 | -0.1402 | 0.0813 | No |
| Subjective Sleep Quality | Sleep Disturbance | -0.0244 | 0.0728 | -0.0972 | -0.1108 | -0.2553 | 0.0294 | No |
| Subjective Sleep Quality | Day Dysfunction | -0.0244 | 0.1311 | -0.1555 | -0.1699 | -0.2951 | -0.0480 | Yes |
| Subjective Sleep Quality | Depression | -0.0244 | 0.0471 | -0.0715 | -0.0753 | -0.2090 | 0.0569 | No |
| Subjective Sleep Quality | Anxiety | -0.0244 | 0.0311 | -0.0555 | -0.0817 | -0.2200 | 0.0501 | No |
| Subjective Sleep Quality | Stress | -0.0244 | 0.1622 | -0.1866 | -0.1999 | -0.3340 | -0.0815 | Yes |
| Sleep Latency | Sleep Duration | 0.0387 | 0.0000 | 0.0387 | -0.0193 | -0.1627 | 0.0698 | No |
| Sleep Latency | Sleep Efficiency | 0.0387 | 0.0000 | 0.0387 | 0.0180 | -0.0819 | 0.1069 | No |
| Sleep Latency | Sleep Disturbance | 0.0387 | 0.0728 | -0.0341 | -0.0657 | -0.1928 | 0.0493 | No |
| Sleep Latency | Day Dysfunction | 0.0387 | 0.1311 | -0.0924 | -0.1248 | -0.2366 | -0.0221 | Yes |
| Sleep Latency | Depression | 0.0387 | 0.0471 | -0.0084 | -0.0302 | -0.1587 | 0.0868 | No |
| Sleep Latency | Anxiety | 0.0387 | 0.0311 | 0.0076 | -0.0366 | -0.1391 | 0.0584 | No |
| Sleep Latency | Stress | 0.0387 | 0.1622 | -0.1235 | -0.1548 | -0.2903 | -0.0259 | Yes |
| Sleep Duration | Sleep Efficiency | 0.0000 | 0.0000 | 0.0000 | 0.0373 | -0.0370 | 0.1586 | No |
| Sleep Duration | Sleep Disturbance | 0.0000 | 0.0728 | -0.0728 | -0.0464 | -0.1549 | 0.0767 | No |
| Sleep Duration | Day Dysfunction | 0.0000 | 0.1311 | -0.1311 | -0.1055 | -0.1992 | 0.0065 | No |
| Sleep Duration | Depression | 0.0000 | 0.0471 | -0.0471 | -0.0109 | -0.1125 | 0.1048 | No |
| Sleep Duration | Anxiety | 0.0000 | 0.0311 | -0.0311 | -0.0173 | -0.1265 | 0.1199 | No |
| Sleep Duration | Stress | 0.0000 | 0.1622 | -0.1622 | -0.1355 | -0.2452 | -0.0227 | Yes |
| Sleep Efficiency | Sleep Disturbance | 0.0000 | 0.0728 | -0.0728 | -0.0837 | -0.1883 | 0.0197 | No |
| Sleep Efficiency | Day Dysfunction | 0.0000 | 0.1311 | -0.1311 | -0.1428 | -0.2416 | -0.0474 | Yes |
| Sleep Efficiency | Depression | 0.0000 | 0.0471 | -0.0471 | -0.0482 | -0.1599 | 0.0559 | No |
| Sleep Efficiency | Anxiety | 0.0000 | 0.0311 | -0.0311 | -0.0546 | -0.1572 | 0.0553 | No |
| Sleep Efficiency | Stress | 0.0000 | 0.1622 | -0.1622 | -0.1728 | -0.2931 | -0.0627 | Yes |
| Sleep Disturbance | Day Dysfunction | 0.0728 | 0.1311 | -0.0583 | -0.0591 | -0.1874 | 0.0594 | No |
| Sleep Disturbance | Depression | 0.0728 | 0.0471 | 0.0257 | 0.0355 | -0.0638 | 0.1438 | No |
| Sleep Disturbance | Anxiety | 0.0728 | 0.0311 | 0.0417 | 0.0291 | -0.1002 | 0.1551 | No |
| Sleep Disturbance | Stress | 0.0728 | 0.1622 | -0.0894 | -0.0891 | -0.2123 | 0.0330 | No |
| Day Dysfunction | Depression | 0.1311 | 0.0471 | 0.0840 | 0.0946 | -0.0265 | 0.2201 | No |
| Day Dysfunction | Anxiety | 0.1311 | 0.0311 | 0.1000 | 0.0882 | -0.0194 | 0.2110 | No |
| Day Dysfunction | Stress | 0.1311 | 0.1622 | -0.0311 | -0.0300 | -0.1514 | 0.0791 | No |
| Depression | Anxiety | 0.0471 | 0.0311 | 0.0160 | -0.0064 | -0.1515 | 0.1278 | No |
| Depression | Stress | 0.0471 | 0.1622 | -0.1151 | -0.1246 | -0.2727 | 0.0165 | No |
| Anxiety | Stress | 0.0311 | 0.1622 | -0.1311 | -0.1182 | -0.2715 | 0.0256 | No |

**Note.** BEI = bridge expected influence. Difference represents BEI for Node 1 minus BEI for Node 2. Confidence intervals are percentile 95% intervals based on 999 successful nonparametric bootstrap samples; one of 1,000 bootstrap samples could not be estimated because the resampled correlation matrix was not positive definite. Intervals including zero indicate that the corresponding BEI point estimates were not clearly distinguishable in the bootstrap difference test.
