# Supplementary material for: Network analysis of physical exercise, sleep quality, and psychological distress among Chinese university students
Source: PLoS One. 2026 Nov 12;21(11):e0362609. doi: 10.1371/journal.pone.0362609 (PMC13759045; doi:10.1371/journal.pone.0362609)
Supplement: Supplementary file 9 [file pone.0362609.s009.docx]

**Table S9. Edge retention frequencies and bootstrap difference tests for cross-domain edges**

**Panel A. Edge retention frequency for all 66 primary-network edges**

| **Node 1** | **Node 2** | **Original edge weight** | **Retention count** | **Retention frequency** |
| --- | --- | --- | --- | --- |
| PE Intensity | PE Duration | 0.2080 | 999/999 | 1.0000 |
| PE Intensity | PE Frequency | 0.0889 | 997/999 | 0.9980 |
| PE Intensity | Subjective Sleep Quality | 0.0000 | 201/999 | 0.2012 |
| PE Intensity | Sleep Latency | 0.0000 | 541/999 | 0.5415 |
| PE Intensity | Sleep Duration | 0.0000 | 197/999 | 0.1972 |
| PE Intensity | Sleep Efficiency | 0.0000 | 209/999 | 0.2092 |
| PE Intensity | Sleep Disturbance | 0.0000 | 92/999 | 0.0921 |
| PE Intensity | Day Dysfunction | 0.0000 | 87/999 | 0.0871 |
| PE Intensity | Depression | -0.0399 | 949/999 | 0.9499 |
| PE Intensity | Anxiety | 0.0000 | 140/999 | 0.1401 |
| PE Intensity | Stress | 0.0000 | 274/999 | 0.2743 |
| PE Duration | PE Frequency | 0.2172 | 999/999 | 1.0000 |
| PE Duration | Subjective Sleep Quality | -0.0805 | 998/999 | 0.9990 |
| PE Duration | Sleep Latency | 0.0000 | 419/999 | 0.4194 |
| PE Duration | Sleep Duration | 0.0000 | 126/999 | 0.1261 |
| PE Duration | Sleep Efficiency | 0.0000 | 258/999 | 0.2583 |
| PE Duration | Sleep Disturbance | 0.0000 | 158/999 | 0.1582 |
| PE Duration | Day Dysfunction | -0.0302 | 893/999 | 0.8939 |
| PE Duration | Depression | 0.0000 | 192/999 | 0.1922 |
| PE Duration | Anxiety | 0.0000 | 395/999 | 0.3954 |
| PE Duration | Stress | -0.0018 | 529/999 | 0.5295 |
| PE Frequency | Subjective Sleep Quality | -0.0374 | 914/999 | 0.9149 |
| PE Frequency | Sleep Latency | 0.0000 | 331/999 | 0.3313 |
| PE Frequency | Sleep Duration | 0.0000 | 276/999 | 0.2763 |
| PE Frequency | Sleep Efficiency | 0.0000 | 307/999 | 0.3073 |
| PE Frequency | Sleep Disturbance | -0.0468 | 948/999 | 0.9489 |
| PE Frequency | Day Dysfunction | 0.0000 | 91/999 | 0.0911 |
| PE Frequency | Depression | 0.0000 | 488/999 | 0.4885 |
| PE Frequency | Anxiety | -0.0206 | 686/999 | 0.6867 |
| PE Frequency | Stress | -0.1105 | 999/999 | 1.0000 |
| Subjective Sleep Quality | Sleep Latency | 0.1655 | 999/999 | 1.0000 |
| Subjective Sleep Quality | Sleep Duration | 0.0000 | 127/999 | 0.1271 |
| Subjective Sleep Quality | Sleep Efficiency | 0.0000 | 125/999 | 0.1251 |
| Subjective Sleep Quality | Sleep Disturbance | 0.1648 | 999/999 | 1.0000 |
| Subjective Sleep Quality | Day Dysfunction | 0.1178 | 999/999 | 1.0000 |
| Subjective Sleep Quality | Depression | 0.0000 | 108/999 | 0.1081 |
| Subjective Sleep Quality | Anxiety | 0.0000 | 341/999 | 0.3413 |
| Subjective Sleep Quality | Stress | 0.0935 | 998/999 | 0.9990 |
| Sleep Latency | Sleep Duration | 0.0406 | 931/999 | 0.9319 |
| Sleep Latency | Sleep Efficiency | 0.0000 | 423/999 | 0.4234 |
| Sleep Latency | Sleep Disturbance | 0.0340 | 864/999 | 0.8649 |
| Sleep Latency | Day Dysfunction | 0.0000 | 115/999 | 0.1151 |
| Sleep Latency | Depression | 0.0000 | 237/999 | 0.2372 |
| Sleep Latency | Anxiety | 0.0387 | 946/999 | 0.9469 |
| Sleep Latency | Stress | 0.0000 | 59/999 | 0.0591 |
| Sleep Duration | Sleep Efficiency | 0.1582 | 999/999 | 1.0000 |
| Sleep Duration | Sleep Disturbance | 0.0000 | 482/999 | 0.4825 |
| Sleep Duration | Day Dysfunction | 0.0000 | 116/999 | 0.1161 |
| Sleep Duration | Depression | 0.0000 | 494/999 | 0.4945 |
| Sleep Duration | Anxiety | 0.0000 | 116/999 | 0.1161 |
| Sleep Duration | Stress | 0.0000 | 361/999 | 0.3614 |
| Sleep Efficiency | Sleep Disturbance | 0.0000 | 279/999 | 0.2793 |
| Sleep Efficiency | Day Dysfunction | 0.0000 | 651/999 | 0.6517 |
| Sleep Efficiency | Depression | 0.0000 | 73/999 | 0.0731 |
| Sleep Efficiency | Anxiety | 0.0000 | 95/999 | 0.0951 |
| Sleep Efficiency | Stress | 0.0000 | 211/999 | 0.2112 |
| Sleep Disturbance | Day Dysfunction | 0.0772 | 991/999 | 0.9920 |
| Sleep Disturbance | Depression | 0.0870 | 999/999 | 1.0000 |
| Sleep Disturbance | Anxiety | 0.0000 | 299/999 | 0.2993 |
| Sleep Disturbance | Stress | 0.0326 | 887/999 | 0.8879 |
| Day Dysfunction | Depression | 0.0000 | 45/999 | 0.0450 |
| Day Dysfunction | Anxiety | 0.0130 | 751/999 | 0.7518 |
| Day Dysfunction | Stress | 0.1483 | 999/999 | 1.0000 |
| Depression | Anxiety | 0.1464 | 999/999 | 1.0000 |
| Depression | Stress | 0.2074 | 999/999 | 1.0000 |
| Anxiety | Stress | 0.1716 | 999/999 | 1.0000 |

**Panel B. Signed bootstrap difference tests comparing Day Dysfunction–Stress with other positive retained cross-domain edges**

| **Compared edge** | **Reference weight** | **Compared weight** | **Difference** | **Bootstrap mean difference** | **95% CI** | **CI excludes zero** |
| --- | --- | --- | --- | --- | --- | --- |
| Subjective Sleep Quality–Stress | 0.1483 | 0.0935 | 0.0548 | 0.0590 | [-0.0143, 0.1331] | No |
| Sleep Latency–Anxiety | 0.1483 | 0.0387 | 0.1096 | 0.1083 | [0.0420, 0.1734] | Yes |
| Sleep Disturbance–Depression | 0.1483 | 0.0870 | 0.0613 | 0.0592 | [-0.0071, 0.1278] | No |
| Sleep Disturbance–Stress | 0.1483 | 0.0326 | 0.1157 | 0.1201 | [0.0502, 0.1870] | Yes |
| Day Dysfunction–Anxiety | 0.1483 | 0.0130 | 0.1353 | 0.1308 | [0.0619, 0.1952] | Yes |

**Panel C. Absolute edge-weight bootstrap difference tests for selected negative cross-domain edges**

| **Compared edge** | **Difference in absolute weights** | **Bootstrap mean difference** | **95% CI** | **CI excludes zero** |
| --- | --- | --- | --- | --- |
| PE Frequency–Stress | 0.0378 | 0.0437 | [-0.0255, 0.1151] | No |
| PE Duration–Subjective Sleep Quality | 0.0678 | 0.0716 | [-0.0041, 0.1388] | No |

Note. Retention frequency is the proportion of successful nonparametric bootstrap samples in which an edge was retained as nonzero after re-estimating the primary network. Of 1,000 requested bootstrap samples, 999 yielded estimable networks; one resample could not be estimated because the resampled correlation matrix was not positive definite. In Panel B, Difference represents the Day Dysfunction–Stress edge weight minus the compared positive cross-domain edge weight. In Panel C, differences are calculated using absolute edge weights. Confidence intervals are percentile 95% bootstrap intervals. Intervals including zero indicate that the corresponding edge weights were not clearly distinguishable under the bootstrap comparison. Retention frequencies and bootstrap intervals describe edge-selection stability and sampling variability and should not be interpreted as conventional significance tests.
