# Supplementary material for: Network analysis of physical exercise, sleep quality, and psychological distress among Chinese university students
Source: PLoS One. 2026 Nov 12;21(11):e0362609. doi: 10.1371/journal.pone.0362609 (PMC13759045; doi:10.1371/journal.pone.0362609)
Supplement: Supplementary file 10 [file pone.0362609.s010.docx]

**Table S10. Gender-network comparison, stability, matched-sample sensitivity, and simulation-based evaluation**

**Panel A. Primary Network Comparison Test (NCT): settings and observed results**

| **Item** | **Specification / result** |
| --- | --- |
| Male / Female sample size | 674 / 926 |
| Network estimator | qgraph::cor_auto() followed by EBICglasso; γ = 0.50; penalize.diagonal = FALSE |
| NCT implementation | NetworkComparisonTest v2.2.3; 1,000 permutations; weighted = TRUE; abs = TRUE; paired = FALSE; binary.data = FALSE; test.edges = TRUE |
| Global-strength statistic | Absolute difference between the two networks' sums of absolute upper-triangle edge weights |
| Male global strength | 2.6920 |
| Female global strength | 2.1908 |
| Global-strength difference | S = 0.5012; p = 0.3806 |
| Permutation interpretation | Approximately 38.1% of label-permuted S statistics were at least as large as the observed S |
| Network-structure difference | M = 0.0914; p = 0.9111 |
| Permutation distribution of S | Mean = 0.4505; SD = 0.3286; median = 0.3840; 2.5th–97.5th percentile = 0.0211–1.2232; range = 0.0011–1.5624 |

**Panel B. Sex-specific network descriptives and case-dropping stability**

| **Metric** | **Male** | **Female** | **Interpretation** |
| --- | --- | --- | --- |
| Non-zero edges | 30 | 25 | Different sparsity was observed |
| Network density | 0.4545 | 0.3788 | Male network was denser descriptively |
| Global strength | 2.6920 | 2.1908 | Descriptive group difference; NCT p = 0.3806 |
| EI CS coefficient | 0.2834 | 0.3607 | Both < 0.50 |
| BEI CS coefficient | 0.3605 | 0.5162 | Male < 0.50; female ≥ 0.50 |
| Top 10 edge differences | 8/10 larger absolute edge weights | 2/10 larger absolute edge weights | Descriptive only; corrected edge tests were not significant |

**Panel C. Equal-sample-size sensitivity analyses**

| **Analysis** | **Sampling** | **Global strength** | **Network structure** |
| --- | --- | --- | --- |
| Single matched-female analysis | Male n = 674; matched female n = 674 | S = 0.6980; p = 0.2238 | M = 0.0963; p = 0.8911 |
| Repeated matched subsampling | 100/100 successful repetitions; 674 women sampled each time | Proportion with global-strength p < 0.05 = 0.010 | Proportion with structure p < 0.05 = 0.000 |

**Panel D. Simulation-based evaluation**

| **Simulation mode** | **Successful / requested replicates** | **Permutations per replicate** | **Global-strength detection proportion** | **Structure detection proportion** |
| --- | --- | --- | --- | --- |
| Observed-effect simulation | 50 / 50 | 500 | 0.060; MC 95% interval 0.000–0.126 | 0.180; MC 95% interval 0.074–0.286 |
| Pooled-null calibration | 50 / 50 | 500 | 0.000 | 0.000 |

Note. EI = expected influence; BEI = bridge expected influence; CS = correlation stability coefficient; MC = Monte Carlo. CS coefficients < 0.50 were treated as insufficient under the prespecified stability criterion. The matched-sample analyses address unequal group sizes but do not constitute equivalence tests. The simulation was an observed-effect sensitivity analysis based on the current male and female network structures, not an a priori power analysis. Because only 50 simulation replicates per mode were run, the detection proportions are imprecise and should be interpreted as coarse supplementary evidence. Non-significant NCT results indicate that differences were not detected under the current sample and analytical framework; they do not establish that the male and female networks are equivalent or identical.
