# Supplementary material for: Network analysis of physical exercise, sleep quality, and psychological distress among Chinese university students
Source: PLoS One. 2026 Nov 12;21(11):e0362609. doi: 10.1371/journal.pone.0362609 (PMC13759045; doi:10.1371/journal.pone.0362609)
Supplement: Supplementary file 11 [file pone.0362609.s011.docx]

**Table S11. Global PSQI score and DASS-21 severity distributions in the study sample**

| **Measure** | **Category** | **n / Statistic** | **%** |
| --- | --- | --- | --- |
| PSQI global score | Mean (SD) | 4.11 (2.13) | - |
|  | Median (range) | 4 (0-12) | - |
|  | PSQI <=5 | 1,217 | 76.1 |
|  | PSQI >5 | 383 | 23.9 |
| Depression | Normal | 1,141 | 71.3 |
|  | Mild | 175 | 10.9 |
|  | Moderate | 212 | 13.3 |
|  | Severe | 45 | 2.8 |
|  | Extremely severe | 27 | 1.7 |
| Anxiety | Normal | 944 | 59.0 |
|  | Mild | 117 | 7.3 |
|  | Moderate | 364 | 22.8 |
|  | Severe | 76 | 4.8 |
|  | Extremely severe | 99 | 6.2 |
| Stress | Normal | 1,372 | 85.8 |
|  | Mild | 93 | 5.8 |
|  | Moderate | 83 | 5.2 |
|  | Severe | 37 | 2.3 |
|  | Extremely severe | 15 | 0.9 |

Note. N = 1,600. Poor sleep quality was defined as a global PSQI score >5. DASS-21 severity bands were based on the multiplied subscale scores. Percentages may not total 100.0 because of rounding.
