# Supplementary material for: Network analysis of physical exercise, sleep quality, and psychological distress among Chinese university students
Source: PLoS One. 2026 Nov 12;21(11):e0362609. doi: 10.1371/journal.pone.0362609 (PMC13759045; doi:10.1371/journal.pone.0362609)
Supplement: Supplementary file 12 [file pone.0362609.s012.docx]

**Figure S1. Gamma = 0.50 versus gamma = 1.00 edge-weight comparison**
